# ASIC1a Induces Excessive Mitophagy and PANoptosis of Chondrocyte by the Inhibition of SIRT3 Mitochondrial Translocation

**DOI:** 10.7150/thno.116712

**Published:** 2025-08-30

**Authors:** Zhuoyan Zai, Xuewen Qian, Yayun Xu, Huifang Lv, Mengjia Hao, Yueming Tao, Lixin Rui, Xiaoyue Zhang, Xiaoqing Peng, Yihao Zhang, Feihu Chen

**Affiliations:** 1 School of Pharmacy, Anhui Medical University, Hefei 230032, China.; 2 Inflammation and Immune Mediated Diseases Laboratory of Anhui Province, Anhui Institute of Innovative Drugs, Anhui Medical University, Hefei 230032, China.; 3 Department of Medicine., University of Wisconsin School of Medicine and Public Health, Madison 53705, WI, USA.; 4 Carbone Cancer Center, University of Wisconsin School of Medicine and Public Health, Madison 53705, WI, USA.; 5 Department of Neurology, The First Affiliated Hospital of Shenzhen University, Shenzhen Second People's Hospital, Shenzhen 518035, China.; 6 School of Public Health, Anhui Medical University, Hefei 230032, China.

**Keywords:** ASIC1a, mitophagy, PANoptosis, SIRT3, oxidative stress

## Abstract

**Rationale:** The death of chondrocytes triggered by extracellular acidification represents a critical factor in the degradation of cartilage tissue and bone, thereby exacerbating the progression of rheumatoid arthritis (RA). Our previous research demonstrated that acid-sensing ion channel 1a (ASIC1a) serves as a key acid sensor mediating the destruction of articular cartilage in RA, which is closely associated with mitochondrial damage of chondrocytes. However, its regulatory mechanism remains unclear.

**Methods:** Cartilage samples from RA patients and collagen-induced arthritis (CIA) rat models were examined to determine the levels of mitophagy and PANoptosis. In parallel, primary rat articular chondrocytes were cultured and subjected to either ASIC1a activation or silencing. Mitochondrial function, mitophagy, and PANoptotic markers were evaluated using immunoblotting, immunofluorescence, and transmission electron microscopy. Additionally, the subcellular distribution of SIRT3 to clarify its role in maintaining mitochondrial homeostasis.

**Results:** We observed a significant increase in the levels of mitophagy and PANoptosis within the cartilage tissue of both RA patients and collagen-induced arthritis (CIA) rat models. Activation of ASIC1a by extracellular acidification triggered mitophagy, ultimately resulting in PANoptosis of chondrocytes. The loss of ASIC1a protected chondrocytes from PANoptosis, thereby alleviating disease progression in CIA rats. Mechanistically, we demonstrated that the transport of SIRT3 from cytoplasm to mitochondria was inhibited upon ASIC1a activation. ASIC1a upregulated calcineurin (CaN) expression, which competitively bound to HSP70, disrupting the SIRT3-HSP70 complex and thereby impairing SIRT3 mitochondrial translocation. The reduced levels of SIRT3 in mitochondria induced mitochondrial dysfunction and excessive mitophagy in primary rat articular chondrocytes, ultimately leading to PANoptosis of chondrocytes. Restoration of SIRT3 improved mitochondrial dysfunction and inhibited excessive mitophagy in the process of ASIC1a-induced PANoptosis of chondrocytes.

**Conclusion:** Our study demonstrated that ASIC1a induces the destruction of articular cartilage through the disruption of the equilibrium between mitochondrial quality control and cell fate. This suggests that ASIC1a is a promising therapeutic target to improve the clinical treatment of RA.

## Introduction

Rheumatoid arthritis (RA) is an autoimmune disease of unknown etiology, predominantly manifested by chronic synovitis and cartilage damage, ultimately resulting in joint deformities and loss of function 1. The damage to articular cartilage represents the primary cause of disability in RA patients, even after first-line drug treatment for RA, where joint inflammation is successfully controlled, articular cartilage continues to deteriorate, leading to a gradual loss of joint function 2, 3. Chondrocytes, the only cell type in cartilage, play a decisive role in the function of this tissue. Therefore, exploring the mechanisms of articular cartilage destruction in RA and identifying potential therapeutic targets to protect chondrocytes are of considerable clinical significance.

The acidification of the joint fluid microenvironment in RA patients is one of the ‌crucial‌ predisposing factors for cartilage damage 3. ASICs are among the acid receptors on the cell membrane, activated by acidification, mediating the influx of Ca^2+^, Na^+^, and other ions and induce various pathological reactions 4. Our previous studies discovered that ASIC1a plays a key role in chondrocyte death in RA 5-7. As a newly recognized form of cell death, PANoptosis, recently recognized form of cell death, is the phenomenon of crosstalk and coordination among pyroptosis, apoptosis, and necroptosis. PANoptosis is regulated by upstream receptors and molecular signaling cascades. Upon sensing pathogen signals, sensor proteins mediate the assembly of proteins involved in pyroptosis, apoptosis, and necroptosis to couple and bind into a multiprotein complex termed PANoptosome, thereby inducing PANoptosis 8-11. However, little is known regarding its specific role in RA cartilage. Our previous studies indicated that ASIC1a induces apoptosis, pyroptosis, and necrosis in rat articular chondrocytes 5, 7, 12, however, it remains largely unknown whether these death-signaling molecules could assemble into PANoptosome and play a regulatory role in RA.

Mitophagy serves as a key quality control mechanism by removing damaged mitochondria and playing a role in apoptosis, necroptosis and pyroptosis 13, 14. Even though mitophagy is often beneficial and reduces mitochondrial damage and cell death, excessive activation also leads to the loss of too many mitochondria, which affects the energy supply of multiple organs and tissues, accelerating cell death and disease progression 15-17. Mitophagy is regulated by a delicate balance, and whether it is beneficial or harmful to diseases depends on the equilibrium between the substrate load and the efficiency of the mitophagy machinery. Therefore, how to maintain mitophagy while avoiding its excessive occurrence has become a key issue. The crucial role of mitophagy in RA treatment is highlighted by increased autophagy levels in the synovial tissue of active RA patients and its correlation with the severity of the disease 18, 19. Our previous studies found that ASIC1a promotes non-selective autophagy and mitochondrial stress in chondrocytes by mediating extracellular Ca^2+^ influx 20, 21. However, the involvement of ASIC1a in mitochondrial function and mitophagy and its role in PANoptosis responses exacerbating disease progression remain poorly defined.

Sirtuin (Sirt1-7) is a highly conserved family of nicotinamide adenine dinucleotide (NAD^+^)-dependent protein deacetylases that are involved in various biological processes 22. The levels of SIRT3, SIRT4, and SIRT5 are significantly reduced in the blood of RA patients 23. As a member of the Sirt family that has attracted considerable attention, SIRT3 plays a critical role in reducing cell damage under various pathological conditions 24, 25. Previous studies have demonstrated that SIRT3 contributes to bone homeostasis, and its deficiency leads to mitochondrial dysfunction, increased oxidative stress, and ultimately accelerated osteoarthritis progression in mice 26-28. A recent study has shown that SIRT3 deficiency induces apoptosis of CD4⁺ T cells and exacerbates antigen-induced arthritis (AIA) in mice 29. However, whether SIRT3 directly functions in cartilage tissue remains unclear.

In the present study, we showed abnormal mitophagy activation in cartilage tissue from RA patients, and demonstrated that ASIC1a induces PANoptosis in chondrocytes by triggering mitochondrial dysfunction and excessive mitophagy. Additionally, we discovered that the inhibition of SIRT3 translocation into mitochondria is characterized as a key regulatory mechanism in the induction of excessive mitophagy and PANoptosis in response to ASIC1a activation. Furthermore, to determine whether ASIC1a influences the progression of RA articular cartilage damage *in vivo*, an adeno-associated virus carrying ASIC1a shRNA was used to infect CIA rats. Our study enhances the understanding of mitophagy's pathological role in RA, and provides evidence for a novel strategy targeting ASIC1a protein in RA treatment.

## Results

### Acidic microenvironment induced chondrocyte PANoptosis by activating ASIC1a

**Figure 1:**
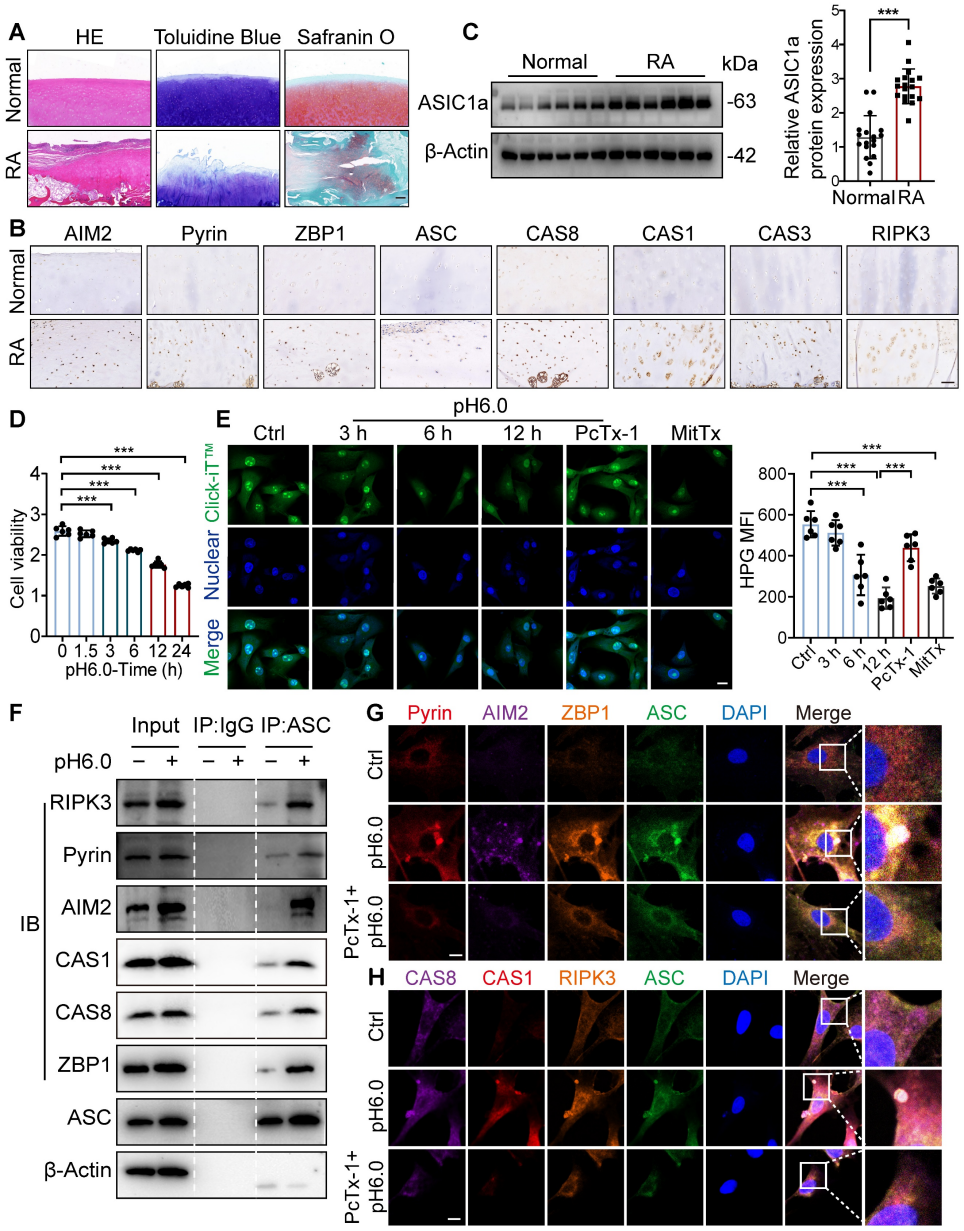
** ASIC1a induces chondrocyte panoptosis and is tightly involved in RA progression. (A)** Representative hematoxylin-eosin staining, toluidine blue staining and Safranin O/fast green staining in cartilage tissues of RA patients and the control group. Scale bar: 200 μm. **(B)** Immunohistochemistry staining for AIM2, Pyrin, ZBP1, ASC, CAS8, CAS1, CAS3 and RIPK3 in cartilage tissues of RA patients and the control group. Scale bar: 100 μm. **(C)** Immunoblot analysis of ASIC1a in cartilages tissues of RA patients and the control group. **(D)** CCK8 analysis of the effects of acid stimulation on the cell viability of chondrocytes. **(E)** Protein synthesis, as determined by L-homopropargylglycine (HPG) mean fluorescence intensity (MFI), in chondrocytes treated with pH6.0 acid, or PcTx-1(100 nM, 30 min before incubation with pH6.0 acid) or MitTx (20 nM). Images are representative of six independent experiments. Scale bar: 20 μm. **(F)** Immunoblot analysis of PANoptosome following immunoprecipitation (IP) with anti-ASC or IgG control antibodies in chondrocytes treated with pH6.0 acid for 12 h. **(G, H)** Immunofluorescence images of chondrocytes after acid stimulation for 12 h. White arrowheads indicate the ASC speck. Scale bar: 10 μm. Images are representative of six independent experiments. Data were presented as mean ± SD and analyzed by Student's t-test or one-way ANOVA. *p < 0.05, **p < 0.01, ***p < 0.001.

To evaluate the contributions of PANoptosis to the progression of RA, we collected cartilage tissues from 18 RA patients who underwent joint replacement surgery and normal cartilage tissues from 18 patients who underwent high amputations due to accidental car accidents. The RA patients exhibited inflammatory infiltrates, loss of glycosaminoglycans and proteoglycans, cartilage destruction, and bone erosion (Figure 1A). Proteins associated with PANoptosis, including AIM2, Pyrin, ZBP1, ASC, CAS8, CAS1, CAS3, and RIPK3 10, 30-32, showed higher expression in the cartilage of RA patients compared to normal tissue (Figure 1B and S1A, B). In parallel, ASIC1a showed higher expression in the cartilage tissue of RA patients than in normal tissue (Figure 1C and S1C), indicating that ASIC1a and PANoptosis are tightly associated with the progression of RA. To investigate the effect of ASIC1a on chondrocyte PANoptosis in RA progression, we cultured primary articular chondrocytes isolated from normal rats and identified the expression of chondrocyte markers via toluidine blue staining of glycosaminoglycan (Figure S1D). The chondrocytes were treated with a pH 6.0 acid solution to mimic the acidic microenvironment in RA joint cavities and activated ASIC1a. Meanwhile, we treated chondrocytes with MitTx (an ASIC1 activator) as the positive control and PcTx-1 (an ASIC1a inhibitor) as the negative control. Cell viability gradually decreased in chondrocytes treated with the acid solution for 1.5 h, 3 h, 6 h, 12 h, and 24 h (Figure 1D). In parallel, acid stimulation impaired protein synthesis and collagen production, significantly decreased the expression of chondrogenic markers, and increased the expression of cartilage degradation markers (Figures 1E and S1E-G), accompanied by the decrease in proliferation and increase in apoptosis (Figures S1H-I). These events were consistent with treatment with MitTx, but were attenuated when chondrocytes were pretreated with PcTx-1 before exposure to pH 6.0, suggesting that acidosis activates ASIC1a and impairs chondrocyte function. Next, we observed that the expression levels of PANoptosis-related proteins are increased following pH6.0 treatment, and found the interactions of ASC with RIPK3, Pyrin, AIM2, CAS1, CAS8 and ZBP1 by immunoprecipitation (Figure 1F), suggesting that ASIC1a has a distinct ability to induce the formation of the PANoptosome. Furthermore, we observed that ASC specks colocalized with AIM2, Pyrin, CAS8, CAS3, RIPK3 and ZBP1 collectively in the same cell 12 h after acid stimulation (Figure 1G-H), indicating the formation of PANoptosome composed of ASC, AIM2, Pyrin, ZBP1, CAS-8, CAS-3 and RIPK3 induced by ASIC1a. These results suggest that ASIC1a leads to chondrocyte dysfunction and PANoptosis. To further evaluate the functional role of PANoptosis in RA progression, we conducted ASC silencing experiments using lentiviral shRNA in acid-stimulated cells (Figure S2A). ASC knockdown led to a significant reduction in the expression of key PANoptosome markers (Figure S2B). In parallel, partial restoration of cell viability and COL2A1 expression were observed under acidic conditions following ASC knockdown (Figure S2C-D). Furthermore, to verify the functional interdependence of apoptotic, pyroptotic, and necroptotic pathways, we inhibited caspase-3 (Z-DEVD-FMK), caspase-1 (VX-765), and RIPK3 (GSK-872) under acidic conditions (pH 6.0). Each inhibitor alone could increase cell viability, whereas simultaneous inhibition of all three pathways produced a more pronounced protective effect (Figure S2E). These findings indicate that PANoptosis plays a contributing role in promoting chondrocyte death and aggravating the progression of RA.

### ASIC1a deficiency attenuated chondrocyte PANoptosis and disease progression

**Figure 2:**
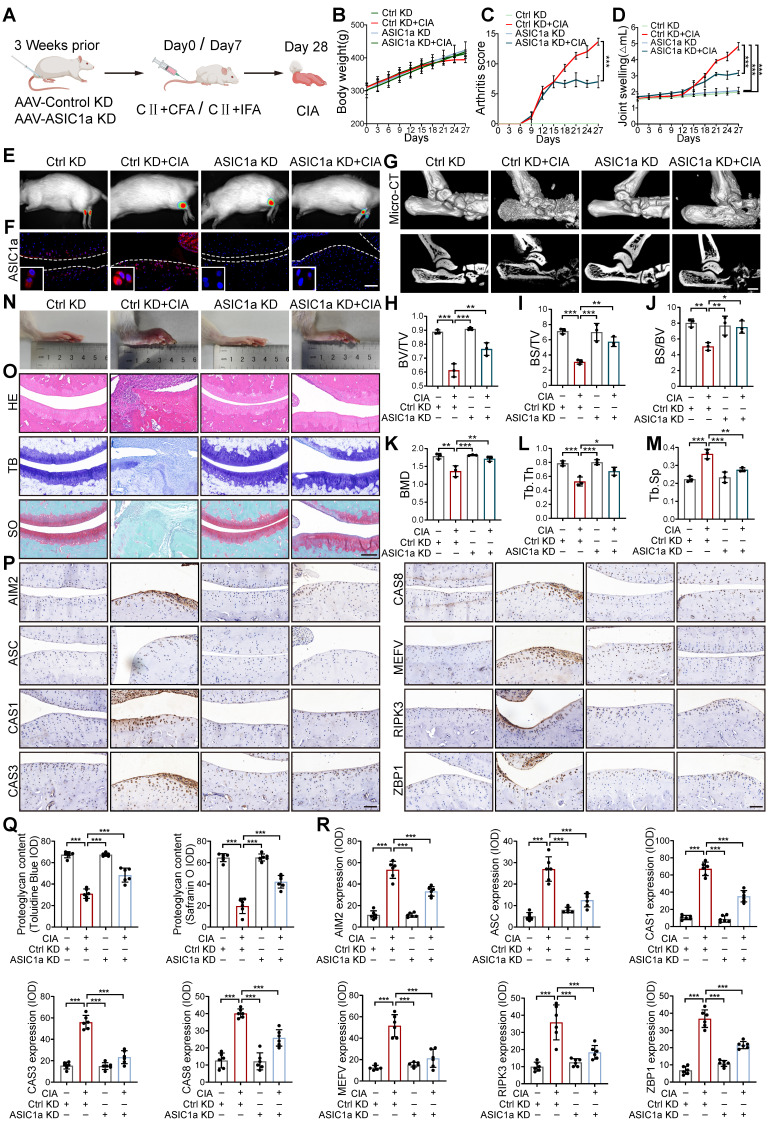
** Cartilage-targeted inhibition of ASIC1a alleviates the pathological progression of CIA. (A)** The experimental design diagram of animal experiment (n = 8 per group). **(B)** The body weight of rats. **(C)** The arthritis score of rats. Traces for CtrlKD and ASIC1aKD groups (non-arthritic controls) overlap with the x-axis due to the absence of arthritis induction (CIA-). **(D)** The joint swelling of rats. **(E)** Representative bioluminescence images of rats were depicted using the IVIS Imaging System (n = 3 per group). **(F)** Immunofluorescence staining for ASIC1a in cartilages of rats. Scale bar: 100 μm. **(G)** Representative images of joints and Micro-CT analysis for the ankles of rats (n = 3 per group). Scale bar: 2 mm. **(H-M)** Quantitative analysis of BV/TV, BS/TV, BS/BV, BMD, Tb.Th and Tb.Sp (n = 3 per group). **(N)** Representative images of rat ankle joints. **(O, Q)** Representative images of H&E, toluidine blue and safranin O/fast green staining of rat cartilages. The proteoglycan content was semi-quantitatively assessed by measuring the integrated optical density (IOD). Scale bar: 200 μm. **(P, R)** Representative images of immunohistochemistry staining for ZBP1, AIM2, RIPK3, ASC, CAS8, CAS3, Pyrin and CAS1 in rat cartilages. Quantification of proteins expression were performed by measuring IOD values. Scale bar: 100 μm. Data were presented as mean ± SD and analyzed by Student's t-test or one-way ANOVA. *p < 0.05, **p < 0.01, ***p < 0.001.

We next tested the contribution of ASIC1a to RA progression. We constructed a silencing model of ASIC1a in rats by local intra-articular injection of adenovirus expressing ASIC1a-specific shRNA (Figure 2A). The efficiency of shASIC1a was verified by *in vivo* imaging, immunofluorescence and western blot, and it had no significant effect on the weight changes of the rats (Figure 2B, E, F and S3A). The results demonstrated that the silence of ASIC1a significantly reduced the joint swelling and arthritis score in CIA rats (Figure 2C-D). Micro-CT scanning, bone and trabecular bone analysis revealed apparent articular cartilage damage and bone destruction in CIA rats, while ASIC1a knockdown effectively ameliorated articular cartilage and bone erosion in CIA rats (Figure 2G-M). Consistently, representative images of rat ankle joints and pathological staining showed severe synovial hyperplasia, inflammatory cell infiltration, cartilage destruction and bone erosion in CIA rats, while these symptoms were significantly improved with ASIC1a deficiency (Figure 2N, O and Q). Immunohistochemical results indicated markedly decreased expression of ZBP1, AIM2, RIPK3, ASC, CAS8, CAS3, Pyrin and CAS1 in cartilage tissues after ASIC1a knockdown (Figure 2P and R). These results suggest that ASIC1a plays a crucial role in inducing PANoptosis, cartilage damage, and aggravating the CIA progression.

To further determine the *in vivo* effects of ASIC1a on cartilage damage in RA, we performed intra-articular injection of different concentrations of ASIC1a specific inhibitor PcTx-1 for treatment in CIA rats. The results showed that PcTx-1 (1 ug/kg and 2 ug/kg) treatment had a significant effect on body weight compared with CIA model group, synovial hyperplasia, inflammatory cell infiltration, joint swelling and arthritis scores were significantly reduced (Figure S4A-E). Micro-CT scanning, bone, and trabecular bone analysis revealed apparent articular cartilage damage and bone destruction in CIA rats, while pharmacological blockade of ASIC1a effectively alleviated articular cartilage and bone erosion in CIA rats (Figure S4F-M). Correspondingly, pathological staining showed that cartilage destruction and bone erosion in CIA rats treated with PcTx-1 (2 ug/kg) were obviously alleviated, similar to the effect of Methotrexate (MTX) (Figure S4N-O). The remarkable accumulation of ZBP1, AIM2, RIPK3, ASC, CAS8, CAS3, Pyrin and CAS1 was shown by the immunohistochemistry, indicating the ongoing PANoptosis in cartilage tissue in CIA model rats, which were improved by PcTx-1 treatment (Figure S4P). Taken together, ASIC1a plays a crucial role in cartilage tissue PANoptosis and CIA pathogenesis, while pharmacological blockade or silencing of ASIC1a effectively prevents CIA progression.

### ASIC1a mediates mitochondrial dysfunction caused by acidification

**Figure 3:**
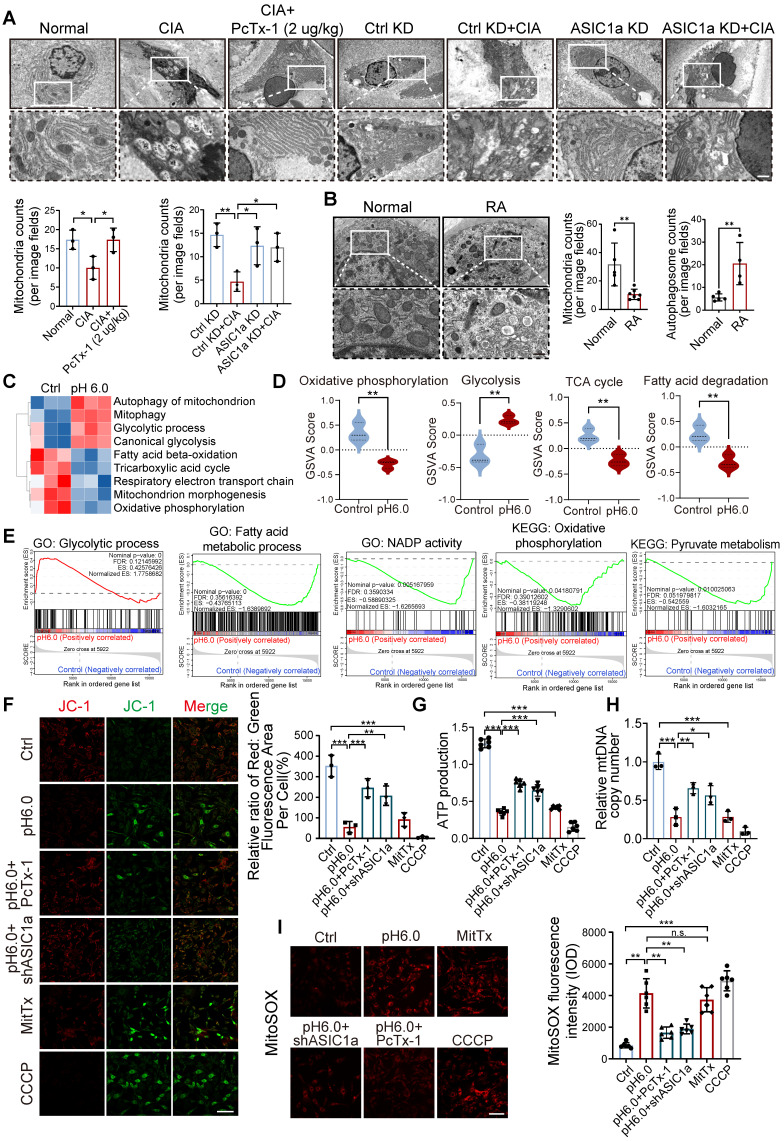
** ASIC1a activation results in severe mitochondrial dysfunction and mitochondrial loss. (A)** Representative TEM images of rat cartilage tissues. Images are representative of three independent experiments. Scale bar: 500 μm. **(B)** Representative TEM images of cartilage tissues from RA patients and the control group. Scale bar: 500 μm. **(C, D)** GSVA results showing the status of mitochondria function, including mitophagy, the tricarboxylic acid cycle, respiratory electron transport chain, glycolytic process, and fatty acid beta-oxidation, identified using KEGG database and GO database. **(E)** GSEA results demonstrating the status of mitochondria function by KEGG, GO and REACTOME database. **(F)** Mitochondrial membrane potential was measured using JC-1 by confocal microscopy in chondrocytes. The ratio of red to green fluorescence intensity was quantified to indicated the level of mitochondrial membrane potential. Images are representative of six independent experiments. Scale bar: 100 μm. **(G)** The intracellular ATP production was measured using an ATP production assay Kit. Data are representative of at least six independent experiments. **(H)** The mtDNA copy number in chondrocytes was measured using a Mitochondrial DNA Copy Number Quantification qPCR Assay Kit. Data are representative of at least three independent experiments. **(I)** MitoSOX levels in chondrocytes were observed by confocal microscopy. Images are representative of six independent experiments. Scale bar: 100 μm. Data were presented as mean ± SD and analyzed by Student's t-test. *p < 0.05, **p < 0.01, ***p < 0.001.

To investigate the cellular structure of cartilage tissue affected by ASIC1a, we used transmission electron microscope (TEM) to assess rat cartilage tissue. We observed severe mitochondrial damage in CIA rats, along with a significant decrease in the total number of mitochondria, while knockdown and pharmacological blockade of ASIC1a effectively alleviated this damage (Figure 3A). Of note, compared to normal cartilage, mitochondria in RA patients also exhibited severe mitochondrial damage, along with a significant decrease in the total number of mitochondria. These results suggest a close association between mitochondrial dysfunction and the development of RA (Figure 3B). We next conducted RNA sequencing on chondrocytes exposed to pH 6.0 for 12h, compared with a normal pH control. Principal component analysis (PCA) comparing acid-stimulated samples to controls showed a clear distinction between the two groups (Figure S5B-C). The annotated genes from GO and KEGG analyses indicated that activation of ASIC1a led to significant enrichment of genes related to mitochondrial functions (Figure S5A and D), GO pathway network further confirmed that mitochondrial function was associated with chondrocyte death (Figure S5E). Subsequently, GSVA using KEGG and GO gene sets revealed that differentially expressed genes under normal and acidified conditions were primarily enriched in mitochondrial metabolism-related pathways, including mitophagy, the tricarboxylic acid cycle, respiratory electron transport chain, glycolytic process, and fatty acid beta-oxidation (Figure 3C-D). Additionally, GSEA based on GO, KEGG databases further demonstrated significant changes in mitochondrial function-related processes. These findings suggest that ASIC1a-induced functional impairment and cell death in chondrocytes are associated with mitochondrial dysfunction (Figure 3E). To further investigate the role of ASIC1a in mitochondrial function, we used an ASIC1a-silencing virus (Figure S6A). Compared to the control group, activation of ASIC1a significantly decreased the mitochondrial membrane potential (Figure 3F), accompanied by lower ATP production and mitochondrial DNA copy number, as well as increased the generation of MitoSOX (Figure 3G-I and S5F). Silencing ASIC1a or treating with PcTx-1 significantly improved these effects. These results suggest that ASIC1a activation leads to mitochondrial dysfunction and loss.

### Excessive mitophagy induces PANoptosis in chondrocytes

**Figure 4:**
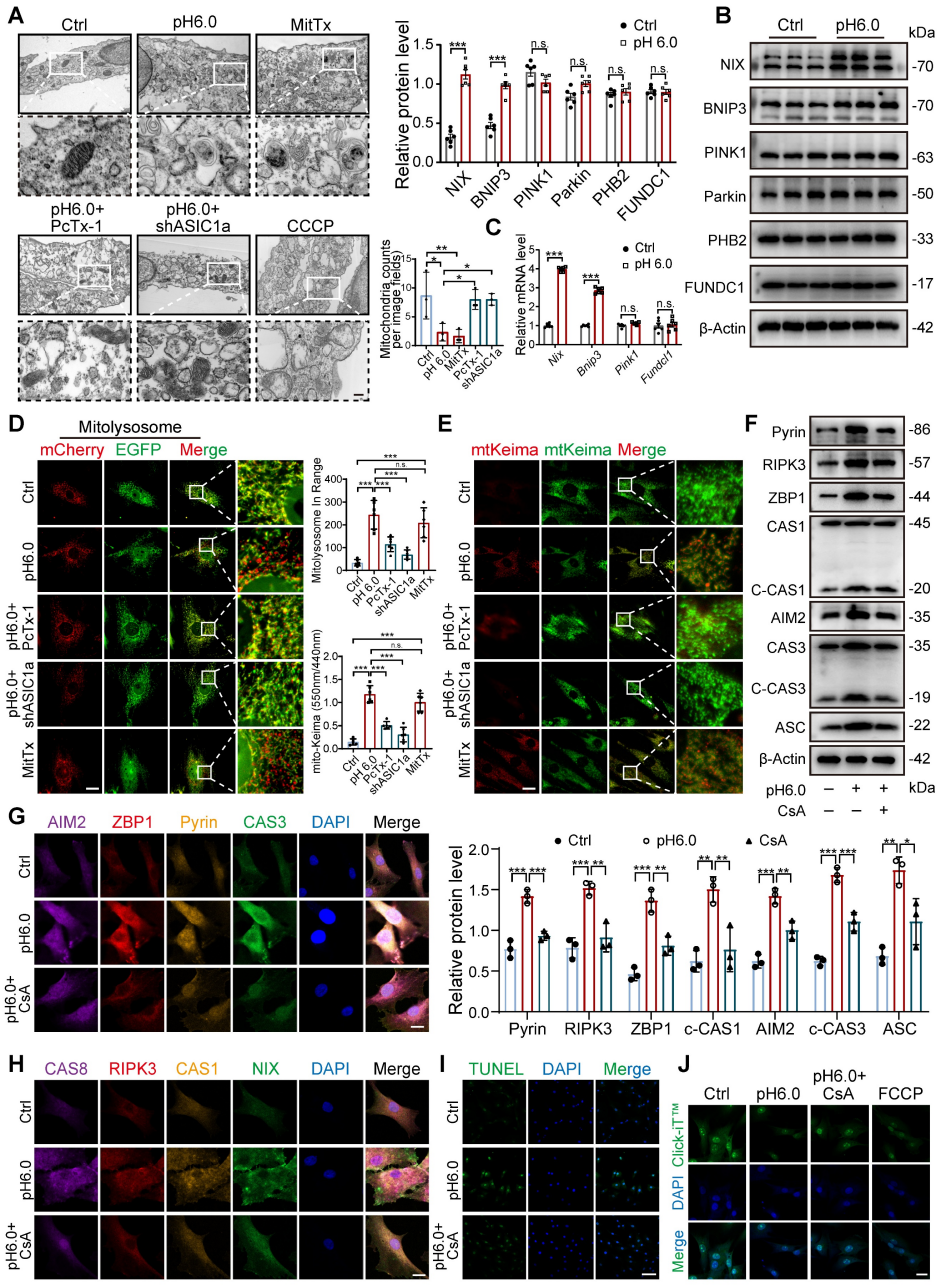
** Excessive mitophagy induces PANoptosis and chondrocyte damage. (A)** The mitochondrial ultrastructure of chondrocytes was detected by TEM. The number of mitochondria was conducted. Images are representative of three independent experiments. Scale bar: 200 μm. **(B)** The protein levels of NIX, BNIP3, PINK1, Parkin, PHB2, FUNDC1 and β-Actin in chondrocytes were determined by western blot. Data are representative of at least six independent experiments. **(C)** The mRNA levels of *Nix*, *Bnip3*, *Pink1* and *Fundc1* in chondrocytes were determined by qRT-PCR. Data are representative of at least six independent experiments. **(D)** Fluorescent dots transfected with COX8-EGFP-mCherry adenovirus were observed by confocal microscopy in chondrocytes. The number of red dots, which indicated the number of mitolysosome, was quantified. Images are representative of six independent experiments. Scale bar: 10 μm. **(E)** Fluorescent dots transfected with mitochondria-targeted Keima (mt-Keima) adenovirus were observed by confocal microscopy in chondrocytes. The relative ratio of red to green fluorescence was quantified. Images are representative of six independent experiments. Scale bar: 10 μm. **(F)** Representative western blot bands of RIPK3, ZBP1, ASC, Pyrin, ZBP1, CAS3, CAS1 and β-actin in chondrocytes. Data are representative of at least three independent experiments. **(G, H)** Representative images of PANoptosome formation of chondrocytes. Images are representative of six independent experiments. Scale bar: 10 μm. **(I)** Representative images of TUNEL assay were applied to detect chondrocyte apoptosis. Images are representative of six independent experiments. Scale bar: 100 μm. **(J)** Representative images of chondrocyte protein synthesis, as determined by L-homopropargylglycine (HPG) mean fluorescence intensity (MFI). Images are representative of six independent experiments. Scale bar: 20 μm. Data were presented as mean ± SD and analyzed by Student's t-test or one-way ANOVA. *p < 0.05, **p < 0.01, ***p < 0.001.

Since not only a decrease in the number and damage of mitochondria but also an increase in the number of autophagic vesicles were observed in RA patients and CIA rats (Figure 3A-B), we speculate that the damaging effect of ASIC1a on cartilage is related to mitophagy. The results revealed higher expression of ASIC1a and LC3B in cartilage tissue from RA patients and CIA rats compared to controls, and that LC3 expression was weakened in the rats in the ASIC1a silenced group. Notably, there was a significant positive correlation between ASIC1a and LC3 expression (Figure S6B-D). Moreover, the LC3-EGFP-mCherry assay demonstrated that ASIC1a activated autophagic flux in chondrocyte, which is consistent with a progressive increase in LC3B-II and a progressive reduction in p62 (Figure S6E-F). Meanwhile, a progressive decrease in mitochondrial proteins COX4I1 and TOM20 was observed, reflecting a decrease in mitochondrial content (Figure S6F). The TEM results revealed that ASIC1a activation caused severe mitochondrial damage and distinct mitophagy in chondrocytes, along with a reduction in the total number of mitochondria (Figure 4A). Subsequently, we examined the protein expression of several key mitophagy receptors, including Parkin, PINK1, NIX, BNIP3, PHB2, and FUNDC1. The results showed a significant increase in NIX and BNIP3 after 12 h of acid treatment, while the expression of Parkin, PINK1, PHB2 and FUNDC1 remained unchanged (Figure 4B). We then selected representative mitophagy genes (*Pink1*,* Nix*, *Bnip3*, and* Fundc1*) for mRNA level analysis, and the transcriptional results were consistent with the corresponding protein expression trends (Figure 4C). Immunohistochemical staining demonstrated higher expression of BNIP3 and NIX in cartilage tissue from RA patients and CIA model group rats compared to controls, and silencing of ASIC1a reduced their expression (Figure S6C-D), further supporting the involvement of NIX and BNIP3 in acid-induced mitophagy. Finally, we infected chondrocytes with a fusion expression adenovirus containing the leader peptide sequence of mitochondrial inner membrane protein Cox8 and the EGFP-mCherry fluorescent tandem sequence, which detects acidic lysosome-engulfed mitochondria as a marker of mitophagy. The results indicated that ASIC1a activated mitophagy in chondrocytes (Figure 4D). We also measured the mitophagic status by using a targeting sequence specifically located in the mitochondrial matrix fused with a pH-dependent keima to form mt-keima. The acidic lysosome causes the excitation of the mtKeima fluorescent protein to change from 440 nm to 590 nm. The results showed more fluorescent dots at 590 nm upon activation of ASIC1a, suggesting that ASIC1a induced mitophagy in chondrocytes (Figure 4E). Silencing or inhibiting ASIC1a improved these phenomena. These results suggest that activation of ASIC1a causes mitochondrial structure damage and excessive mitophagy.

To further evaluate whether excessive mitophagy mediates chondrocyte PANoptosis, we performed additional mitophagy blockade experiments using the mitophagy inhibitor cyclosporin A (CsA) as a protective agent. We found that blockade of mitophagy significantly decreased the levels of PANoptosome (Figure 4F-H). Futhermore, blockade of mitophagy by CsA effectively enhanced protein synthesis ability and reduced apoptosis levels compared to the acid treatment group (Figure 4I-J). These results suggest that inhibiting excessive mitophagy improves ASIC1a-induced PANoptosis in chondrocytes.

### ASIC1a inhibits the translocation of SIRT3 to mitochondria

**Figure 5:**
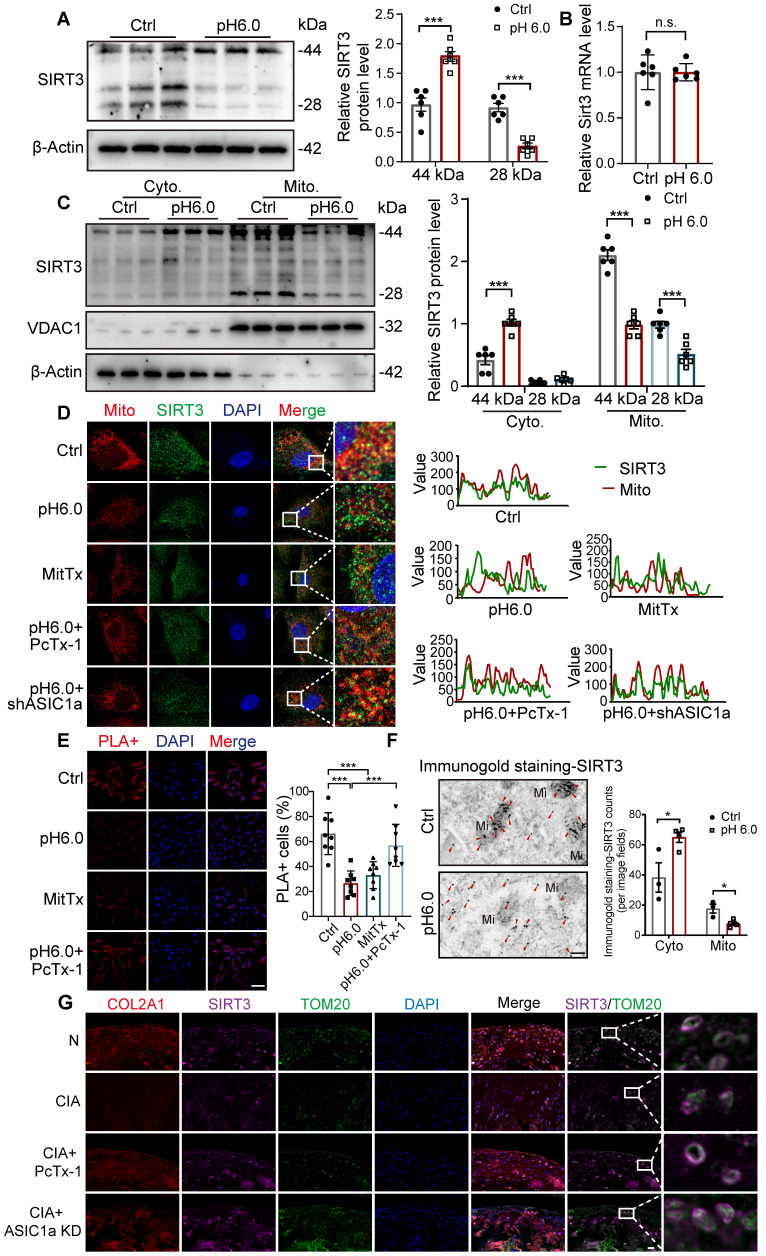
** Activation of ASIC1a leads to the inhibition of SIRT3 transport to mitochondria. (A)** The protein level of SIRT3 was determined by western blot in chondrocytes. **(B)** The mRNA level of SIRT3 was determined by qRT-PCR. **(C)** Immunoblotting of SIRT3 in mitochondrial and cytosolic fractions was performed. **(D)** The colocalization of mitochondria and SIRT3 was observed. The colocalization level of mitochondria with SIRT3 per cell was quantified. Images are representative of six independent experiments. Scale bar: 10 μm. **(E)** Confocal images of the PLA of SIRT3 and TOMM20 were obtained. The percentages of PLA^+^ cells were calculated. Images are representative of six independent experiments. Scale bar: 100 μm. **(F)** Representative images of immunogold labeling of SIRT3. Mi, mitochondria; Red arrow, SIRT3. Data are representative of at least three independent experiments. Scale bar: 200 μm. **(G)** Immunofluorescence staining for COL2A1, SIRT3, TOM20 in rats. Scale bar: 20 μm. Data were shown as mean ± SD and analyzed by Student's t-test or one-way ANOVA. *p < 0.05, **p < 0.01, ***p < 0.001.

Sirtuins, including SIRT3, SIRT4, and SIRT5, are involved in key processes that support mitochondrial health, dynamics, and function 24, 33-35. We detected the expression of SIRT3, SIRT4, and SIRT5 in chondrocytes. For SIRT3, the full-length protein (~44 kDa) is present in the cytosol and is proteolytically processed in the mitochondrial matrix into an active ~28kD form. Notably, the expression of the full-length SIRT3 protein (~44 kDa) increased, while the expression of 28KD product decreased after activation of ASIC1a (Figure 5A). There was no significant change in the expression of SIRT4 and SIRT5 proteins (Figure S7A), suggesting that SIRT3 is a downstream regulator of ASIC1a activity. Then we found that the mRNA levels of *Sirt3* showed no significant change after acid stimulation for 12 hours (Figure 5B), indicating that SIRT3 transcription is not regulated by ASIC1a. Next, we separated mitochondrial and cytosolic protein fractions. Strikingly, the result showed that in the cytosolic fractions, only the 44 kDa form of SIRT3 was detected, and its expression increased after acid stimulation. In contrast, in the mitochondrial lysates, both forms of SIRT3 were detected, and their expression decreased after acid stimulation (Figure 5C). These results suggest that activation of ASIC1 leads to decreased expression of SIRT3 in mitochondria and increased expression in the cytoplasm. Considering the potential localization of SIRT3 to mitochondria, we detected the impact of ASIC1a on the distribution of SIRT3 by measuring the co-localization of SIRT3 with mitochondria. The results showed a reduction in the co-localization of SIRT3 with the MitoTracker after activation of ASIC1 (Figure 5D). Furthermore, we performed in situ proximity ligation assays (PLAs) to more precisely examine the translocation of SIRT3. The results showed that acid stimulation reduced the spatial approximation of SIRT3 to VDAC1, a mitochondrial outer membrane protein (Figure 5E). In addition, we confirmed this localization of SIRT3 to mitochondria and cytoplasm in chondrocytes using immunogold staining. We found that SIRT3 was accumulated in the cytoplasm while decreasing in mitochondria upon activation of ASIC1a (Figure 5F). These findings suggest that ASIC1a induces mitochondrial dysfunction and PANoptosis by inhibiting the translocation of SIRT3 to mitochondria rather than by regulating the expression of SIRT3. Moreover, compared to normal rats, the mitochondrial localization of SIRT3 was significantly reduced in the cartilage tissue of CIA model rats. Treatment with an ASIC1a-specific inhibitor-PcTx-1 notably improved SIRT3 mitochondrial localization in CIA rats (Figure 5G), confirming that ASIC1a also suppresses SIRT3 mitochondrial trafficking *in vivo*, which is consistent with our findings *in vitro*.

### ASIC1a disrupts HSP70-SIRT3 interaction to impair SIRT3 mitochondrial translocation and function

**Figure 6:**
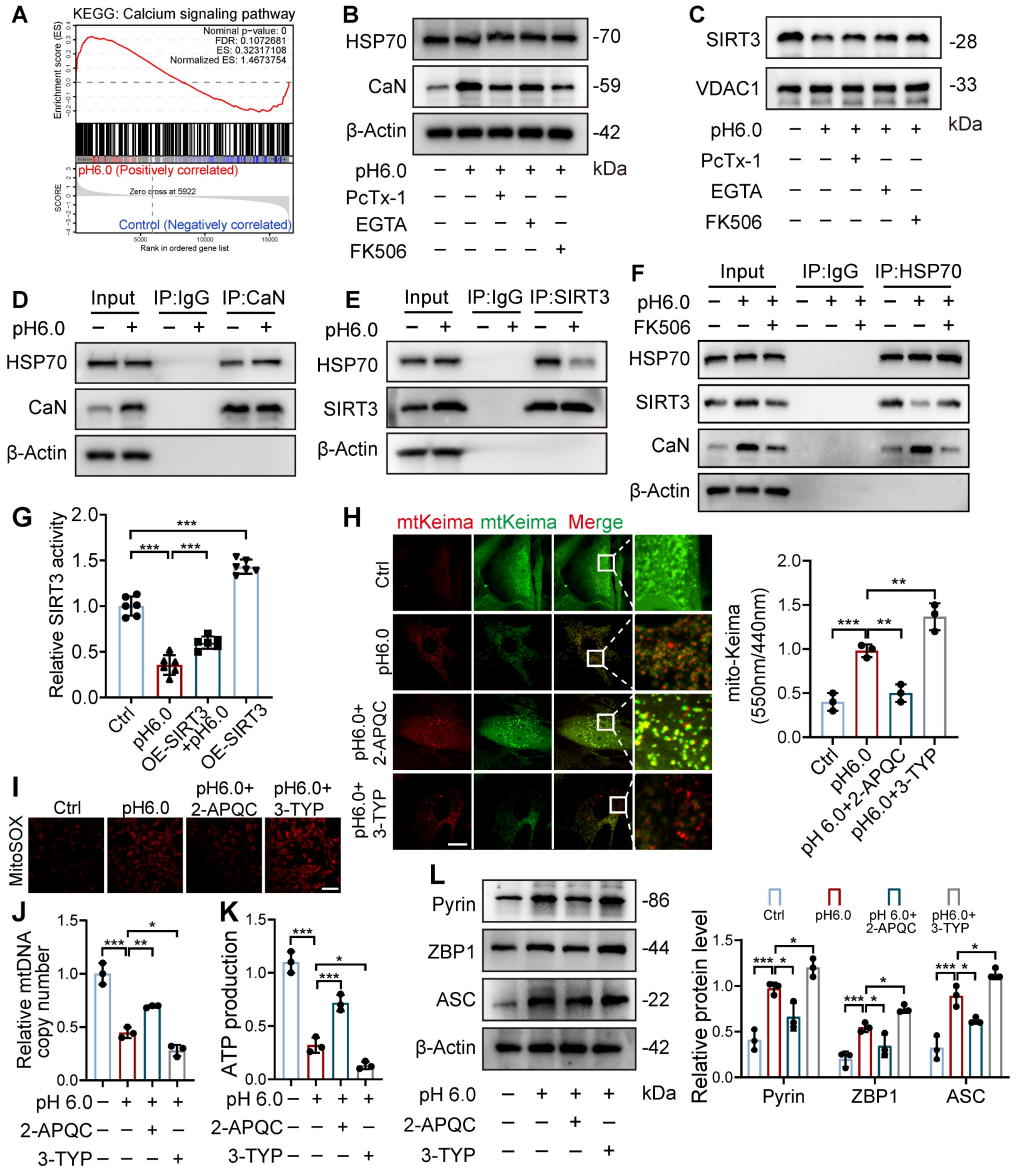
** ASIC1a-CaN-HSP70 axis disrupts SIRT3 mitochondrial import and function. (A)** GSEA results demonstrating the status of calcium signaling pathway by KEGG database. **(B)** The protein levels of HSP70, CaN and β-actin in chondrocytes were determined by western blot. **(C)** The protein levels of SIRT3 and VDAC1 in chondrocytes were determined by western blot. **(D)** Co-IP analysis of the interaction between CaN and HSP70. **(E)** Co-IP analysis of the interaction between SIRT3 and HSP70. **(F)** Co-IP analysis of the interaction between HSP70, SIRT3 and CaN. **(G)** SIRT3 enzymatic activity in chondrocytes was measured using a fluorometric SIRT3 activity assay kit. **(H)** Fluorescent dots transfected with mt-Keima adenovirus were observed by confocal microscopy in chondrocytes. The relative ratio of red to green fluorescence was quantified. Scale bar: 10 μm. **(I)** MitoSOX levels in chondrocytes were observed by confocal microscopy. Scale bar: 100 μm. **(J)** The mtDNA copy number in chondrocytes was measured using a Mitochondrial DNA Copy Number Quantification qPCR Assay Kit. **(K)** The intracellular ATP production was measured using an ATP production assay Kit. **(L)** The protein levels of Pyrin, ZBP1, ASC and β-actin in chondrocytes were determined by western blot. Data were presented as mean ± SD and analyzed by Student's t-test or one-way ANOVA. *p < 0.05, **p < 0.01, ***p < 0.001.

Next, we investigated how ASIC1a influences the mitochondrial translocation of SIRT3. We observed that the calcineurin (CaN) signaling pathway in chondrocytes was significantly activated after acid stimulation, and treatment with the calcium chelator EGTA or the CaN inhibitor Tacrolimus (FK506) markedly reduced CaN expression. Notably, both EGTA and FK506 significantly enhanced mitochondrial SIRT3 levels (Figure 6A-C, S7B-C). SIRT3 contains a mitochondrial targeting sequence, and HSP70 plays a crucial role in mitochondrial protein import by stabilizing precursor proteins, preventing misfolding, and facilitating translocation. Our results showed that ASIC1a activation did not significantly affect HSP70 expression (Figure 6B and S7B). Interestingly, acid stimulation significantly increased the interaction between CaN and HSP70 while decreasing HSP70-SIRT3 binding, whereas treatment with a CaN inhibitor alleviated these changes by reducing HSP70-CaN interaction and enhancing HSP70-SIRT3 binding (Figure 6D-F). These findings suggest that activated CaN competitively binds to HSP70, and that this enhanced CaN-HSP70 interaction disrupts the formation of the cytosolic SIRT3-HSP70 complex, thereby reducing SIRT3 mitochondrial translocation.

To further explore whether the downstream effects of ASIC1a are dependent on SIRT3's deacetylase activity, we conducted a SIRT3 enzymatic activity assay, which revealed that ASIC1a activation significantly downregulated SIRT3 enzymatic activity, and SIRT3 overexpression partially restored its activity (Figure 6G). Notably, SIRT3 overexpression alone increased both its enzymatic activity and mitochondrial localization, demonstrating that enhanced mitochondrial targeting of SIRT3 led to corresponding elevation of its enzymatic activity (Figure 6G and S7D-E). Then we used both the SIRT3 activator 2-APQC and its inhibitor 3-TYP, and found 2-APQC significantly ameliorated ASIC1a-mediated mitophagy levels, reduced mitochondrial superoxide release, and decreased the expression of PANoptosis-related proteins, while 3-TYP exhibited synergistic effects with pH 6.0 treatment, leading to exacerbated mitophagy and aggravated mitochondrial dysfunction (Figure 6H-L). These findings suggest that ASIC1a reduces SIRT3 enzymatic activity by inhibiting its mitochondrial translocation, thereby leading to mitochondrial dysfunction and PANoptosis.

### SIRT3 translocation inhibition leads to excessive mitophagy and PANoptosis

**Figure 7:**
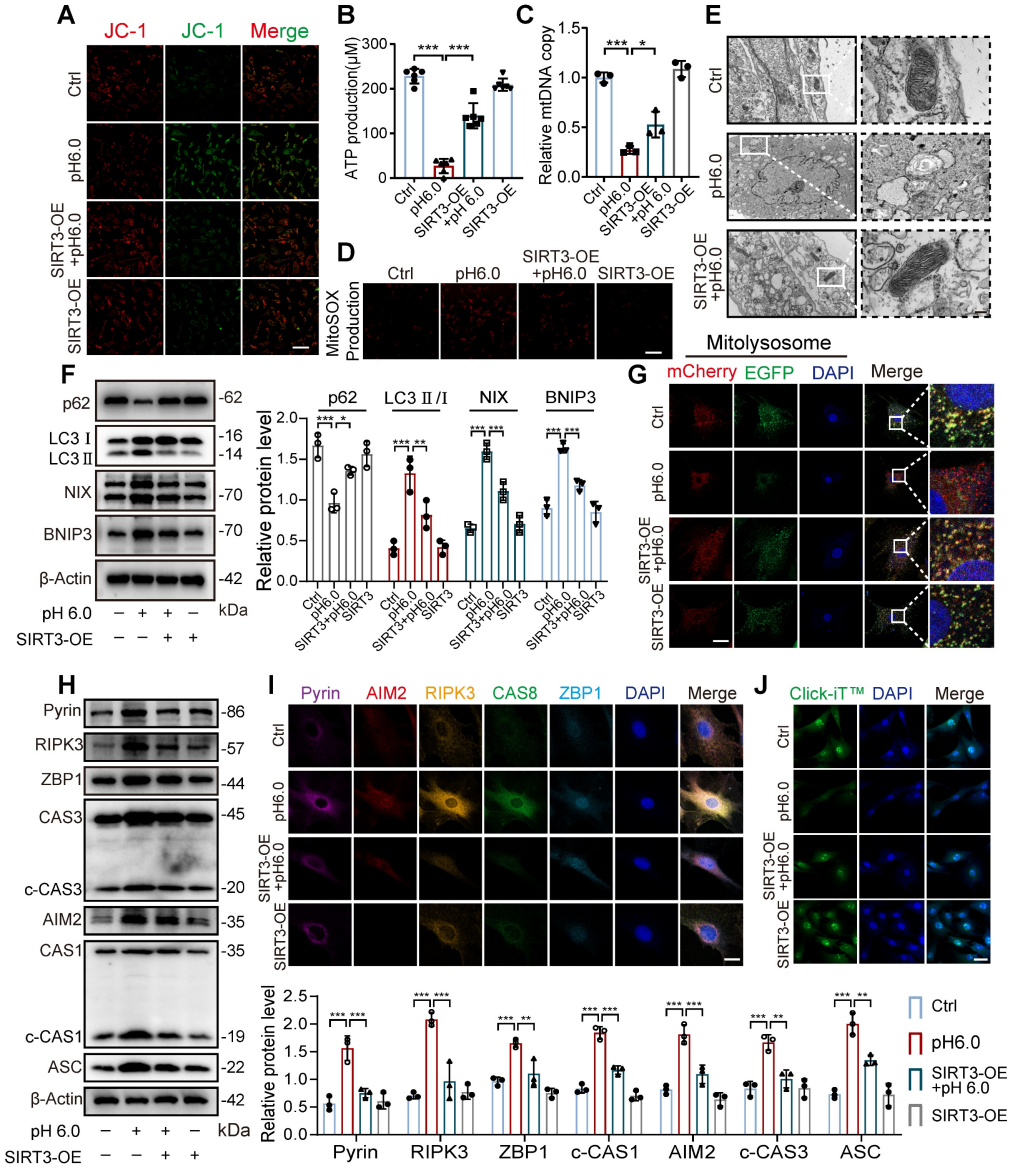
** Restoration of SIRT3 protects against excessive mitophagy and PANoptosis in chondrocyte. (A)** After overexpression of SIRT3 in chondrocytes, mitochondrial membrane potential was measured using JC-1 by confocal microscopy. The ratio of red to green fluorescence intensity was quantified to indicate the level of mitochondrial membrane potential. Images are representative of six independent experiments. Scale bar: 100 μm. S7. **(B)** Intracellular ATP production was measured using an ATP production assay Kit. Data are representative of at least six independent experiments. **(C)** The mtDNA copy number in chondrocytes was measured using a Mitochondrial DNA Copy Number Quantification qPCR Assay Kit. Data are representative of at least three independent experiments. **(D)** MitoSOX level in chondrocytes was observed by confocal microscopy. Images are representative of six independent experiments. Scale bar: 100 μm. **(E)** The chondrocyte mitochondrial ultrastructure was detected by TEM. Images are representative of three independent experiments. Scale bar: 200 μm. **(F)** The protein levels of p62, LC3, NIX, BNIP3, and β-actin in chondrocytes were determined by western blot. Data are representative of at least three independent experiments. **(G)** Fluorescent dots transfected with COX8-EGFP-mCherry adenovirus were observed by confocal microscopy in chondrocytes. The number of red dots, which indicated the number of mitolysosome, was quantified. Images are representative of six independent experiments. Scale bar: 10 μm. **(H)** The protein levels of RIPK3, ZBP1, ASC, CAS1, CAS3, Pyrin, AIM2, and β-actin in chondrocytes were determined by western blot. Data are representative of at least three independent experiments. **(I)** After overexpression of SIRT3 in chondrocytes, the PANoptosome map of chondrocytes exposed to acidosis. Images are representative of six independent experiments. Scale bar: 10 μm. **(J)** Protein synthesis, as determined by L-homopropargylglycine (HPG) mean fluorescence intensity (MFI). Images are representative of six independent experiments. Scale bar: 100 μm. Data were shown as mean ± SD and analyzed by Student's t-test or one-way ANOVA. *p < 0.05, **p < 0.01, ***p < 0.001.

Given that SIRT3 overexpression enhances its enzymatic activity and mitochondrial localization, we further investigated whether restoring SIRT3 levels through overexpression could improve the effects induced by ASIC1a. First, we found that compared to normal chondrocytes, stimulation with pH 6.0 acid in SIRT3-overexpressing chondrocytes ameliorated the ASIC1a activation-induced decrease in mitochondrial membrane potential, ATP levels and mtDNA copy number (Figure 7A-C and S7F). Meanwhile, the restoration of SIRT3 attenuated ASIC1a induced oxidative stress (Figure 7D and S7G). Moreover, mitochondrial structural damage was also ameliorated (Figure 7E and S7H). These results suggest that ASIC1a-induced mitochondrial dysfunction is alleviated by the overexpression of SIRT3. Next, we conducted mitophagy analysis, acid stimulation in SIRT3-overexpressing cells upregulated p62 expression while downregulating the expression of LC3, NIX, and BNIP3, when compared to acid-treated normal cells (Figure 7F). Restoration of SIRT3 significantly reduced ASIC1a-induced accumulation of mitolysosome puncta and efficiently reduced the level of excessive mitophagy (Figure 7G and S7I). Consistently, the LC3-EGFP-mCherry assay showed that overexpression of SIRT3 reduced the increased autophagy levels in chondrocytes caused by ASIC1a activation (Figure S7J). In addition, restoration of SIRT3 decreased the expression level of PANoptosome (Figure 7H-I) and improved chondrocyte function (Figure 7J and S7K). Altogether, these results suggest that the restoration of SIRT3 levels improved mitochondrial dysfunction and inhibited excessive mitophagy during ASIC1a-induced functional damage and PANoptosis of chondrocytes.

## Discussion

Reducing cartilage injury is a crucial yet often overlooked aspect of protecting articular function in RA patients. Acid-sensing ion channel 1a (ASIC1a) serves as a key acid sensor mediating the destruction of chondrocyte and articular cartilage in RA, a process closely associated with mitochondrial dysfunction in chondrocytes 21. Restoring mitochondrial function could be an effective therapeutic approach for RA 36-38. In this study, we discovered heightened mitophagy and PANoptosis activation in cartilage samples obtained from RA patients compared with normal people. We found that ASIC1a activation induces mitochondrial dysfunction and excessive mitophagy, ultimately leading to chondrocyte PANoptosis. We also identified a novel mechanism whereby ASIC1a disrupts SIRT3 mitochondrial localization and deacetylase activity through promote CaN-HSP70 interaction, establishing a direct link between ASIC1a and both mitochondrial dysfunction and PANoptosis. The silencing or inhibition of ASIC1a restores SIRT3 transport into mitochondria, improves mitochondrial function, inhibits excessive mitophagy, and rescues chondrocyte PANoptosis, alleviating disease progression in CIA rats. These results uncover a new mechanism of chondrocyte death and provide a key target for the clinical treatment of RA.

The acidified microenvironment of the joint cavity promotes chondrocyte death and cartilage destruction via activation of ASIC1a, which in turn leads to joint dysfunction. The formation of this acidified environment is the result of the interplay between inflammatory responses and local circulatory impairment. The enclosed anatomical structure of the joint cavity further facilitates the accumulation of lactate within synovial fluid, resulting in a significant decrease in intra-articular pH 39-41. Previous research has shown that ASICs, particularly ASIC1a, play key roles in the progression of RA, mediating cartilage injury through coordinating multiple forms of chondrocyte death. Extracellular acidosis induces chondrocyte apoptosis by activating ASIC1a and upregulating CAS3, CAS9, calpain, and calcineurin, while blockade of ASIC1a reduces apoptosis-related gene expression, rescuing chondrocytes from apoptosis 5, 42, 43. Moreover, the expression of ASC, caspase-1, NLRP3 and other inflammatory or pyroptosis-related factors are upregulated in arthritis rats and ASIC1a-activated chondrocytes, and IL-1β simultaneously enhances ASIC1a-induced chondrocyte apoptosis 7, 44. Furthermore, it has been reported that the C-terminal of ASIC1a recruits RIP1, which induces RIP1 phosphorylation and neuronal death, exacerbating brain injury 45. Our previous study showed that blocking ASIC1a reduces cartilage damage by inhibiting the activities of RIP1 and RIP3 in arthritis rats 12. Although apoptosis, pyroptosis, and necrosis were observed in chondrocytes, inhibiting a single mode of death did not provide significant cartilage protection, suggesting that the intricate crosstalk and coordination among these death patterns. In recent years, PANoptosis, a novel form of programmed cell death regulated by the PANoptosome complex and integrating apoptosis, pyroptosis, and necroptosis, has attracted increasing attention. Here, we found that PANoptosis-related proteins (AIM2, CAS8, CAS1, CAS3, Pyrin, RIPK3, ASC and ZBP1) are upregulated in RA cartilage compared to normal tissues. We also found that acidosis-driven PANoptosome activation, leading to PANoptosis. This process impairs chondrocyte function and contributes to joint dysfunction in RA. These results not only provide new mechanistic insights but also help to clarify the relationship between our earlier work and the current findings, offering a more integrated view of how ASIC1a contributes to chondrocyte damage and RA pathology.

Mitophagy dysregulation has been proposed as a key factor in inducing PANoptosis 46, 47. In active RA patients, the autophagy levels in synovial tissue, circulating lymphocytes, monocytes, and granulocytes are significantly higher than in OA patients, and correlate with anti-cyclic citrullinated peptide antibodies levels 48-50. Additionally, elevated ROS induces oxidative DNA damage and mtDNA mutations, linking mitochondrial dysfunction to disease progression 37. Similarly, high levels of ROS are found in RA cartilage, reflecting mitochondrial damage within the tissue 21. However, the direct contribution of mitophagy to the cartilage damage in RA patients requires further investigation. Our findings provide evidence that an increase in the number of autophagosomes in the cartilage of RA patients, accompanied by a reduction in the number of mitochondria. Activated ASIC1a suppresses chondrocyte oxidative phosphorylation and the TCA cycle while increasing glycolysis, resulting in decreased ATP production, an increase in mtROS release, and a reduction in both mitochondrial membrane potential and mtDNA copy number. While synovial cells exhibit increased autophagy that may support inflammation and cell survival, excessive mitophagy in chondrocytes may reflect a maladaptive response to mitochondrial stress, contributing to cartilage degeneration. This suggests that autophagy in RA is highly context-dependent, contributing differently to various tissues at different stages of RA. Previous reports have shown that deletion of autophagy-related gene ATG7 alleviates cartilage destruction in experimental models, supporting the notion that dysregulated mitophagy may be detrimental in certain contexts 51. Our data further delineate a temporal trajectory in which mitophagy transitions from a homeostatic safeguard to a pathological driver under acidic stress. In the early phase following ASIC1a activation, mitophagy preserved mitochondrial integrity and maintained chondrocyte anabolic activity, consistent with its canonical role in mitochondrial quality control. It is widely accepted that mitophagy serves as a compensatory mechanism to eliminate dysfunctional mitochondria and maintain ROS balance 52-54. However, excessive activation of mitophagy leads to a significant reduction in the number of mitochondria, which accelerates disease progression 15, 17, 55. Under conditions of sustained ASIC1a hyperactivation, both oxidative phosphorylation and electron transport chain function are impaired, while glycolysis is upregulated. This metabolic reprogramming paradoxically leads to increase ROS accumulation and subsequent activation of the PANoptosome complex. Recent studies have proposed that the opening of the mitochondrial permeability transition pore (mPTP) triggers mitochondrial fragmentation and initiates autophagy, leading to excessive and non-selective mitochondrial clearance, a detrimental phenomenon 56. Consistent with this, our findings show that treatment with the mPTP inhibitor cyclosporine A (CsA) significantly reduces ASIC1a-mediated excessive mitophagy and mitigated chondrocyte damage. In this context, restoring mitophagy homeostasis in chondrocytes emerges as a promising strategy to prevent PANoptosis, with SIRT3 identified as a key regulatory factor in this process.

SIRT3, an important mitochondrial deacetylase, plays a crucial role in regulating various aspects of mitochondrial function and protecting mitochondria from damage 24, 28, 57. Although acetyltransferases such as GCN5L1 and ACTA1 are present in mitochondria and are capable of catalyzing lysine acetylation on mitochondrial proteins, recent studies have revealed that non-enzymatic acetylation reactions driven by acetyl-CoA can also result in widespread protein acetylation within the mitochondria. In addition, SIRT3, a NAD⁺-dependent deacetylase, is widely recognized as a major regulator of mitochondrial protein acetylation. Evidence from previous studies indicates that mitochondrial protein acetylation is markedly increased in SIRT3-deficient mice, underscoring the critical role of SIRT3 in maintaining mitochondrial acetylation homeostasis 58-59. Our results demonstrate that a SIRT3 activator ameliorates ASIC1a-mediated mitophagy and mitochondrial dysfunction, while its inhibitor exacerbates these effects, confirming the functional dependence of ASIC1a's pathological outcomes on SIRT3 enzymatic activity. The restoration of SIRT3 alleviates the effects of ASIC1a on mitochondrial and chondrocyte function by inhibiting non-ubiquitin-dependent pathway receptors NIX and BNIP3. Interestingly, there is a reciprocal interaction between ROS and SIRT3 in the regulation of the BNIP3/NIX. On one hand, mitochondrial ROS elevation activates the transcription factor HIF, leading to increased expression of BNIP3 and NIX 60-64. On the other hand, mitochondrial SIRT3 reduces ROS accumulation by deacetylating key proteins such as isocitrate dehydrogenase 2 (IDH2), superoxide dismutase 2 (SOD2), and catalase (CAT), and genetic deletion of the SIRT3 could increase ROS production, which in turn enhances HIF-1 stability and expression of HIF-dependent genes 65-67. These findings highlight the critical role of SIRT3 as a deacetylase in maintaining mitochondrial homeostasis. Furthermore, we reveal that ASIC1a regulates the mitochondrial translocation of SIRT3, which represents a key mechanism by which ASIC1a modulates mitophagy.

In response to acidosis, we demonstrate that SIRT3 is accumulated in the cytoplasm and nucleus, while its levels in mitochondria are significantly reduced. ASIC1a activation reduces mitochondrial import of SIRT3, thereby impairing its enzymatic activity. SIRT3 is expressed in two different forms: ~28KD short form, which is exclusively localized in mitochondria, and the ~44kDa long form (full-length form), which is found both in mitochondria and other cellular compartments. SIRT3 is translated on cytosolic ribosomes and subsequently requires the assistance of molecular chaperones, such as HSP70, to cooperate with the mitochondrial membrane translocation system for import 68-70. SIRT3 contains a mitochondrial targeting sequence that directs its translocation into mitochondria. HSP70 stabilizes the SIRT3 precursor, prevents its misfolding, and facilitates its passage through translocases such as Tom20 into the mitochondria. Subsequently, the 44 kDa precursor form of SIRT3 is cleavage into the 28 kDa mature form within mitochondria, where it exerts its deacetylase activity 71, 72. Studies have shown that activation of CaN can bind to HSP70 and maintain its stability. Our previous work demonstrated that acidification induces activation of the NFAT4 transcription factor in synovial fibroblasts, with NFAT family members being canonical downstream substrates of CaN 73. In this study, transcriptomic sequencing further confirmed that calcium signaling pathways are significantly activated in chondrocytes following acidification. This activation markedly promotes the interaction between CaN and cytosolic HSP70, while simultaneously reducing the interaction between HSP70 and SIRT3, thereby impairing the mitochondrial import of SIRT3. SIRT3 overexpression increases both its enzymatic activity and mitochondrial localization, demonstrating that enhanced mitochondrial targeting of SIRT3 leads to a corresponding elevation in its activity. This enhanced SIRT3 expression alleviates the adverse effects of ASIC1a on mitophagy and chondrocyte function. While some studies have primarily focused on the effects of mitochondrial calcium overload on metabolism and ROS production, our data support the notion that changes in cytosolic signaling networks can impact mitochondrial function, potentially preceding alterations in mitochondrial calcium levels.

In conclusion, this study elucidates a previously unrecognized regulatory axis involving ASIC1a, SIRT3, mitophagy, and PANoptosis in the pathogenesis of RA. We demonstrate that ASIC1a activation impairs SIRT3 function, leading to excessive mitophagy and triggering PANoptotic cell death in chondrocytes. This sequence of events highlights a pivotal link between mitochondrial dysfunction and chondrocyte injury, providing mechanistic insight into the progression of cartilage destruction in RA. By uncovering this pathway, our findings not only advance the understanding of chondrocyte death mechanisms but also suggest that targeting ASIC1a-SIRT3 signaling may offer promising therapeutic potential in RA treatment. Importantly, while our data establish a compelling connection between ASIC1a activity and chondrocyte degeneration, further validation through *in vivo* rescue experiments is warranted. Specifically, the use of lentiviral-mediated overexpression of SIRT3 in animal models, particularly under conditions of ASIC1a activation, would provide critical evidence to support the therapeutic relevance of modulating this pathway. Such studies would not only confirm the protective role of SIRT3 but also offer a preclinical basis for future drug development.

## Materials and Methods

### Patients and tissues

RA cartilage tissues were obtained from 18 patients who met the 2010 ACR diagnostic criteria and underwent joint replacement surgery. Normal cartilage tissues were obtained from 18 patients who underwent high amputation at the First Affiliated Hospital of Anhui Medical University and the Second Hospital of Anhui Medical University. This study has been reviewed and approved by the Human Research Ethics Committee of Anhui Medical University (20210331, Hefei, China), and informed consent was obtained from all participants.

### Animal experiments

SD male rats (8 weeks, 230 ± 20 g) were purchased from Vital River (Beijing, China). The adeno-associated virus 9 (AAV9) expressing ASIC1a-specific shRNA was manufactured by Hanbio Biotechnology (Shanghai, China). To construct the ASIC1a-silencing model, we performed local intra-articular injections of AAV9 into both the left and right knee joints of rats, using 30 μL per joint at a titer of 1×10¹² v.g./mL. The primer sequence for ASIC1a shRNA is provided in Table S1. Rats were randomly divided into nine groups (6 rats/group): control group, CIA model group, PcTx-1 (0.5, 1 and 2 μg/kg) groups, shControl + CIA group, shControl + control group, shASIC1a + CIA group, shASIC1a + control group, and MTX group. Complete Freund's adjuvant (CFA) mixed with bovine collagen II (CII) (Chondrex Inc., USA) was administered, and a booster injection of incomplete Freund's adjuvant (IFA) mixed with CII was given on day 7 to establish the CIA rat model. Arthritis severity was assessed based on a standardized arthritis scoring (AS) system, with detailed criteria provided in Figure S4. All experimental protocols described in this study were reviewed and approved by the Animal Experimental Ethics Review Committee of Anhui Medical University (LLSC20210469, Hefei, China).

### Primary cell culture

Primary rat articular chondrocytes (RACs) were obtained from male Sprague-Dawley (SD) rats weighing 140-160 g, purchased from Vital River (Beijing, China). Knee articular cartilage tissues were isolated under sterile conditions. The tissues were minced into small pieces in phosphate buffered saline (PBS). Subsequently, 0.25% type II collagenase was added to digest the tissues at 37 ℃ in a 5% CO_2_ atmosphere for 6 h. Next, the cells were filtered using a filter membrane and cultured at 37 ℃ in a 5% CO_2_ atmosphere. Chondrocytes from the 3rd to 7th generations were used for experiments. To simulate the acidic microenvironment observed in RA joint cavity, a pH-adjusted culture medium was prepared. The pH of the complete culture medium was adjusted to 6.0 using sterile-filtered 1 M HCl under aseptic conditions and verified using a calibrated pH meter. Cells were exposed to acidified culture medium (pH 6.0) for different time to simulate an acidic environment, and the control group was treated in parallel with fresh standard (non-acidified) medium for the same duration.

### Protein synthesis and proximity ligation assays (PLA)

Protein synthesis was performed using the Click-iT^TM^ L-homopropargylglycine (HPG) Alexa Fluor 488 Protein Synthesis Assay Kit (Thermo Fisher Scientific) in accordance with the manufacturer's instructions. Proximity ligation assays were analyzed using Duolink reagents kits (DUO92002, DUO92004, DUO92008, Sigma, USA) as per the manufacturer's instructions.

### Immunogold labeling and transmission electron microscopy (TEM)

Chondrocytes and tissues were fixed in 4% paraformaldehyde at room temperature for 30 min and subsequently fixed in 2.5% glutaraldehyde at 4 ℃ for 12 h. The fixed samples were washed and incubated in 0.1 M phosphate buffer (pH 7.2) for 10 min, then cut into 70 nm ultrathin sections and counterstained for TEM observation.

For immunogold labeling, sections were blocked with 0.1% BSA for 20 min. The samples were incubated with the primary antibody (rabbit anti-SIRT3) for 4 h at room temperature, followed by incubation with the second antibody (Colloidal gold AffiniPure goat Anti-rabbit IgG (H+L)). Ordinary sections were further fixated in 1% osmium tetroxide for 2 h after washing in PBS, dehydrated in a graded series of ethanol for 20 min, transferred to a mixture of alcohol and iso-amyl acetate for incubated for 1 h, and then dehydrated and counterstained for TEM observation.

### Multiplex immunofluorescence analysis

The immunostaining was performed by Pentuple-Fluorescence kit (RS0039, Immunoway). Briefly, the sample was incubated in multiple rounds of staining, sequentially with different primary antibodies and separate fluorescent tyramide signal amplification systems. EDTA-based antigen retrieval was used in between rounds of tyramide signal amplification to remove the antibody from the previous round. DAPI was utilized as a nuclear stain. The samples were analyzed using a confocal microscope (Zeiss, Germany). The information of antibodies used in this experiment are listed in Table S2.

### Immunoblot analysis

Chondrocyte mitochondrial proteins were extracted using a mitochondrial protein extraction kit (Beyotime, China). For total protein, cells were lysed with RIPA buffer containing protease inhibitor (MCE, China), and the supernatant was collected after centrifugation. Proteins were separated via electrophoresis through 7.5% - 12.5% polyacrylamide gels. After transferring the proteins onto PVDF membranes (Milli-pore, IPVH00010), the membranes were incubated with 5% skim milk, followed by incubation with different antibodies. The next day, the membranes were washed with TBST for three times and incubated with the appropriate horseradish peroxidase (HRP)-conjugated secondary antibodies for 1.5 h. The enhanced chemiluminescence kit (ECL-plus, Thermo Fisher Scientific) was used to image the membranes. The gray values were quantified using the ImageJ software. The information of the antibodies used in this experiment are listed in Table S2.

### mtDNA and ATP

Mitochondrial DNA was isolated using the Rat Mitochondrial DNA Fluorescence Probe PCR Detection Kit (FT0325A/B, EnzyValley, China) according to the manufacturer's instructions. A fluorescence PCR detector was used for detection. Relative quantitation was carried out using the ΔΔCt method. The enhanced ATP Assay Kit (Beyotime, China) was used to measure adenosine 5'-triphosphate, and a chemiluminescence instrument was used to detect the relative light unit (RLU) value.

### MitoSOX and JC-1

The JC-1 mitochondrial membrane potential detection kit (Beyotime, China) and the mitochondrial superoxide staining kit (Beyotime, China) were used to measure mitochondrial membrane potential (Δψm) and mitochondrial superoxide (MitoSOX), respectively. Chondrocyte were inoculated in glass-bottomed laser confocal dishes and subjected to different treatments, then incubated with JC-1 staining solution or MitoSOX staining solution for 30 min in dark conditions. Fluorescence intensity was measured using a confocal microscope (Zeiss, Germany). J-monomers were measured under conditions of 490nm excitation and 530 nm emission, and J-aggregates were measured under the conditions of 525 nm excitation and 590 nm emission. MitoSOX was detected under conditions of 510 nm excitation and 580 nm emission.

### Mito-Keima mitophagy analysis

Chondrocytes were inoculated in laser confocal dishes with a glass bottom and infected with an adenovirus harboring the mito-Keima, and grown for 36 hours. The Keima protein expressed by mKeima is localized in the mitochondrial matrix. When a mitophagy body fuses with an acidic lysosome, the fluorescence signal of the Keima protein shifts from green to red. This conversion of fluorescence signal quantitatively reflects the occurrence and progression of mitophagy. After different treatments, a laser confocal microscope (Zeiss, Germany) was used to measure the level of mitophagy. Mito-keima underwent dual-excitation ratiometric pH measurements at 440 nm (pH 7) and 550 nm (pH 4) lasers with 620 nm emission filters.

### Quantitative real‑time polymerase chain reaction (q-PCR)

Total RNA was extracted from chondrocytes using Trizol reagent (AG Scientific, China), the RNA was reverse-transcribed into cDNA using Evo M-MLV RT Premix (AG Scientific, China). qPCR was performed using the SYBR Green Premix Pro Taq HS qPCR kit (AG Scientific, China) according to the manufacturer's instructions. All the primer sequences used are shown in Table S3.

### RNA sequencing

mRNA with poly(A) tails in total RNA is enriched using Oligo(dT)-conjugated magnetic beads. The mRNA is fragmented into segments of approximately 300 bp via ion fragmentation. Segments of 300 bp are selected for further processing. Using the fragmented RNA as a template, the first strand of cDNA is synthesized with random hexamers and reverse transcriptase. The second strand of cDNA is then synthesized using the first strand as a template. After library construction, PCR amplification enriches the library fragments. Subsequently, the library is size-selected to achieve a final fragment size of 450 bp. The quality of the library is assessed using an Agilent 2100 Bioanalyzer, and both the total concentration and effective concentration of the library are determined. Based on the effective concentration and required sequencing depth, libraries containing unique index sequences (to distinguish post-sequencing data from each sample) are pooled proportionally. The pooled library is diluted to a uniform concentration of 2 nM, and single-stranded libraries are generated through alkaline denaturation. Following RNA extraction, purification, and library preparation, the samples undergo paired-end (PE) sequencing on the Illumina platform using next-generation sequencing (NGS) technology.

### Immunohistochemical staining and Immunofluorescence staining

RA patient cartilage joint tissues and rat cartilage joint tissues were fixed, soaked, and embedded in paraffin, followed by sectioning. Hematoxylin and eosin (H&E) staining, Safranine/Fast Green staining, and toluidine blue staining were conducted using kits (Beyotime, China) in accordance with the manufacturer's protocols. For immunohistochemical staining, the tissue sections were heated, fixed, deparaffinized, rehydrated, and subjected to antigen repair. Subsequently, the sections were permeabilized with 0.5% Triton X-100 (Beyotime, China) in PBS and blocked with 5% BSA for 1 h, followed by incubation with different primary antibodies. The next day, the sections were incubated with the appropriate secondary antibodies for 1 h. A digital pathological section scanner (3DHISTECH, Hungary) was used to examine the sections. For immunofluorescence staining, tissue or cell slides were treated using 5% BSA for 1 h after being fixed in 4% paraformaldehyde, and then incubated overnight at 4 ℃ with different antibodies. The next day, the slides were incubated with the corresponding fluorescent-labeled secondary antibody (FITC, Boster, 1:200) for 1 h in the dark. Tissue sections were captured using a digital pathological section scanner (3DHISTECH, Hungary), and cell sections were photographed using a laser confocal microscope (Zeiss, Germany). The information of the antibodies used in these experiments are listed in Table S2.

### Co-immunoprecipitation (Co-IP)

We used the Classic IP/CoIP Kit (BioLinkedin, L-1004) following the manufacturer's protocol. Cell lysates were prepared and incubated with ASC antibody (Abcam, ab309497, diluted 1:30) for immunoprecipitation (IP). The negative control was rabbit IgG (SouthernBiotech, 0111-01, 1:50). The lysate-antibody mixture was incubated at room temperature at 4°C overnight, followed by the addition of protein A/G magnetic beads and further incubation at 4°C overnight. Unbound proteins were removed through two washes with IP Lysis/Wash Buffer. Finally, the antigen-antibody complex was eluted from the beads for subsequent analysis.

### Statistical analysis

GraphPad Prism 10 was used to analyze the data, which were presented as mean ± standard deviation (SD) from at least three independent biological repeats. T-tests (for two groups) or a one-way analysis of variance (ANOVA, for multiple groups) were used to compare groups to determine whether they differed significantly. P < 0.05 was regarded as significant, *p < 0.05, **p < 0.01, and ***p < 0.001 indicating the levels of significance.

## Supplementary Material

Supplementary figures and tables.

## Abbreviations

AIM2: Inflammasome 2
ASC: apoptosis - associated speck - like protein containing a CARD
ASIC1a: acid-sensing ion channel 1a
ATP: Adenosine Triphosphate
BNIP3: BCL2 Adenovirus E1B 19kDa Interacting Protein 3‌‌
CAS1: Caspase 1
CAS3: Caspase 3
CAS8: Caspase 8
CIA: collagen-induced arthritis
Cox8: cytochrome c oxidase subunit 8
CsA: cyclosporin A
EDU: 5-Ethynyl-2'-deoxyuridine
EGFP: Enhanced Green Fluorescent Protein‌‌
HE: Hematoxylin-Eosin Staining
LC3: Microtubule-associated protein 1 light chain 3
mCherry: monomeric Cherry fluorescent protein‌
MitoSOX: mitochondrial superoxide
mtDNA: mitochondrial deoxyribonucleic acid‌
mtKeima: mKeima-Red-Mito
NIX: Nip-like protein X
RA: rheumatoid arthritis
PcTx-1: Psalmotoxin-1
PINK: PTEN induced putative kinase 1
RIPK3: Receptor Interacting Protein Kinase 3
PLAs: proximity ligation assays
ROS: reactive oxygen species
SIRT3: Sirtuin-3‌
TEM: Transmission electron microscopy
TUNEL: Terminal deoxynucleotidyl Transferase-mediated dUTP Nick End Labeling
VDAC1: Voltage-Dependent Anion Channel 1
ZBP1: Z-DNA binding protein 1
