# Supplementary material for: ASIC1a Induces Excessive Mitophagy and PANoptosis of Chondrocyte by the Inhibition of SIRT3 Mitochondrial Translocation
Source: Theranostics. 2025 Aug 30;15(18):9623–42. doi: 10.7150/thno.116712 (PMC12486255; doi:10.7150/thno.116712)
Supplement: Supplementary file 1 — Supplementary figures and tables. [file thnov15p9623s1.pdf]

Supplementary Material:

ASIC1a induces excessive mitophagy and PANoptosis of chondrocyte by the inhibition of SIRT3 mitochondrial translocation

Zhuoyan Zai[a, b, c, d #], Xuewen Qian[a, b #], Yayun Xu[e #], Huifang Lv[a, b], Mengjia Hao[a, b], Yueming Tao[a, b], Lixin Rui[c, d], Xiaoyue Zhang[f], Xiaoqing Peng[a, b*], Yihao Zhang[f *], Feihu Chen[a, b*]

[a]School of Pharmacy, Anhui Medical University, Hefei 230032, China; [b]Inflammation and Immune Mediated Diseases Laboratory of Anhui Province, Anhui Institute of Innovative Drugs, Anhui Medical University, Hefei 230032, China; [c]Department of Medicine and [d]Carbone Cancer Center, University of Wisconsin School of Medicine and Public Health, Madison 53705, WI; [e]Department of Neurology, The First Affiliated Hospital of Shenzhen University, Shenzhen Second People's Hospital, Shenzhen 518035, China; [f]School of Public Health, Anhui Medical University, Hefei 230032, China

***Corresponding authors:**
Dr Xiaoqing Peng, Email: pengxiaoqing@ahmu.edu.cn
Dr Yihao Zhang, Email: ahmuzyh@ahmu.edu.cn
Dr Feihu Chen, Email: chenfeihu@ahmu.edu.cn
#These authors contributed equally to this work.

This file includes:

Number of figures: 7, Figure S1-S7

Number of tables: 4, Table S1-S4

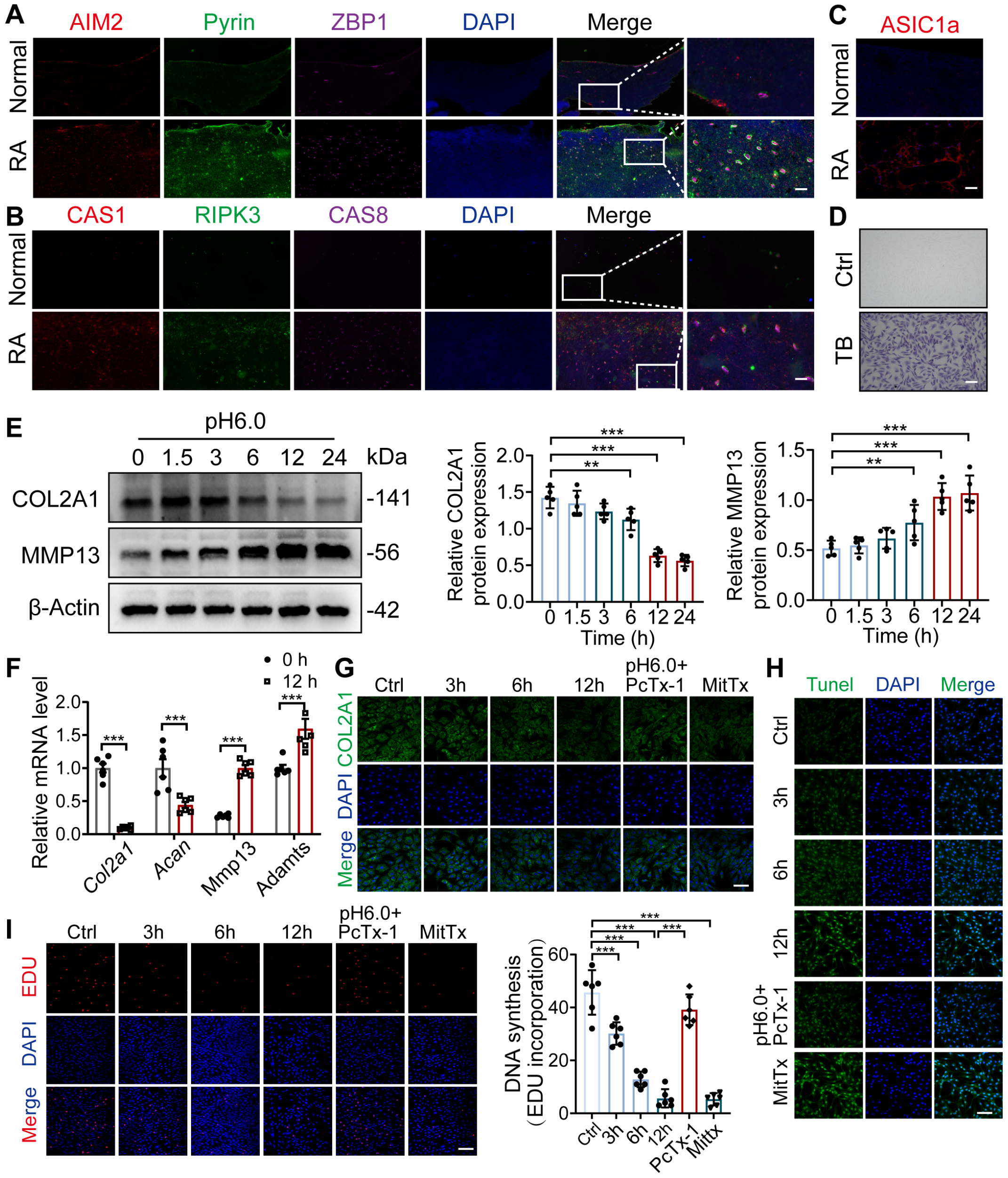

Figure S1. Activation of ASIC1a inhibits chondrocyte function. (A, B) Immunofluorescence images for AIM2, Pyrin, ZBP1, CAS8, RIPK3 and CAS1 in cartilage tissues of RA patients and the control group. Scale bar: 100 μm. (C) Immunofluorescence staining for ASIC1a in cartilage tissues of RA patients and the control group. Scale bar: 100 μm. (D) Biosynthesis of glycosaminoglycan evidenced by toluidine blue staining of rat articular chondrocytes. (E) Immunoblot analysis of COL2A1 and MMP13 in chondrocytes treated with pH6.0 acid for different times. (F) The mRNA levels of *Col2a1*, *Acan*, *Mmp13* and *Adamts* in chondrocytes treated with pH6.0 acid for 12h. Data are representative of at least six independent experiments. (G) COL2A1 immunostaining of chondrocytes treated with pH6.0 acid, PcTx-1(100 nM, 30 min before incubation with pH6.0 acid) or MitTx (20 nM). Images are representative of six independent experiments. Scale bar: 100 μm. (H) TUNEL assay was applied to detect apoptosis of chondrocytes treated with pH6.0 acid, PcTx-1, and

MitTx. Images are representative of six independent experiments. Scale bar: 100 μm. (I) EdU assay was applied to analyze the proliferation ability in chondrocytes treated with pH6.0 acid, PcTx-1, or MitTx. Images are representative of six independent experiments. Scale bar: 200 μm. Data were presented as mean ± SD and analyzed by Student's t-test or one-way ANOVA. *$p < 0.05$, **$p < 0.01$, ***$p < 0.001$.

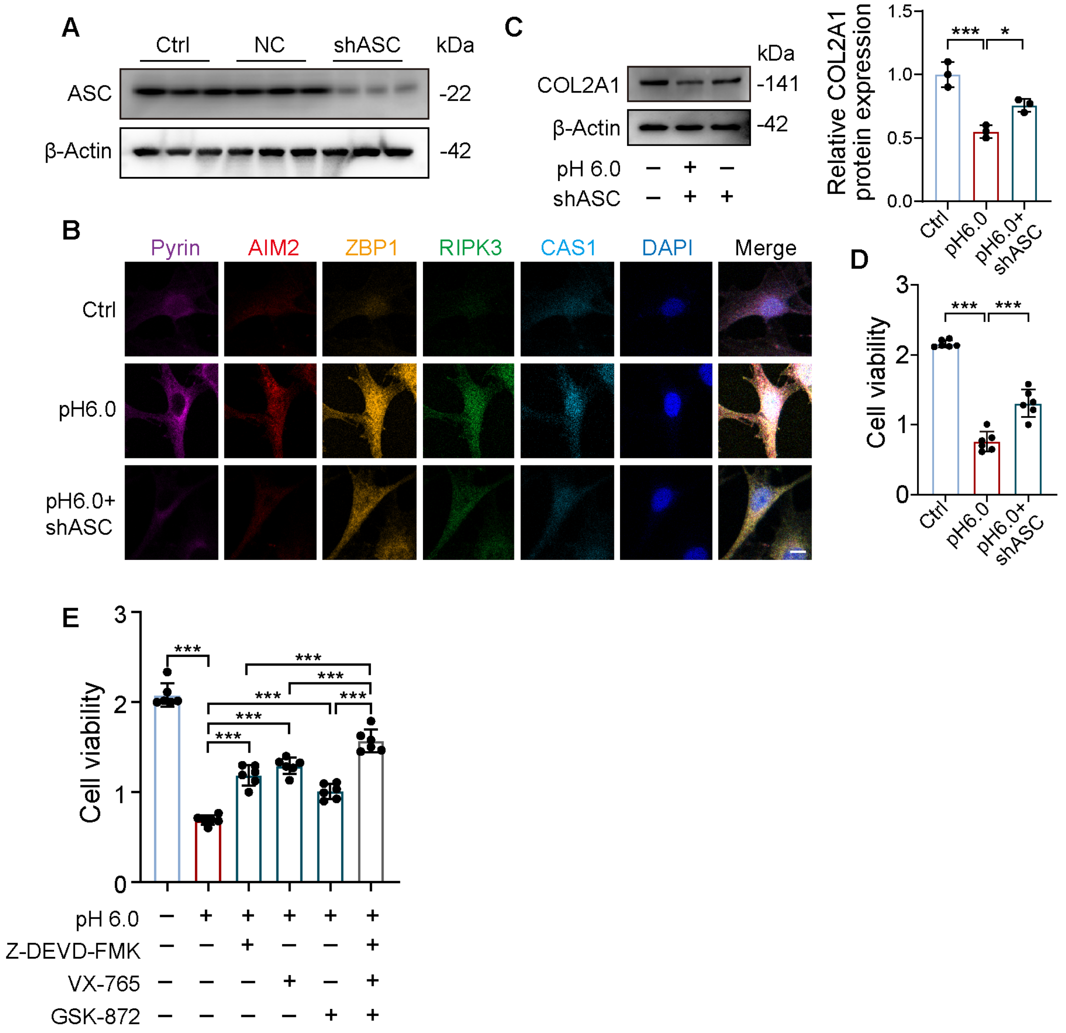

Figure S2 ASC knockdown attenuates PANoptosis activation and chondrocyte damage under acidic conditions. (A) The ASC and β-actin protein levels were determined by western blot in shASC chondrocytes. (B) Representative images of PANoptosome formation of chondrocytes. Scale bar: 10 μm. (C) Immunoblot analysis of COL2A1 in shASC chondrocytes under acidic condition. (D) CCK8 analysis the cell viability in shASC chondrocytes under acidic condition. (E) CCK-8 analysis of cell viability under acidic condition with treatment of Z-DEVD-FMK, VX-765, GSK-872. Data were presented as mean ± SD and analyzed by one-way ANOVA. *$p < 0.05$, **$p < 0.01$, ***$p < 0.001$.

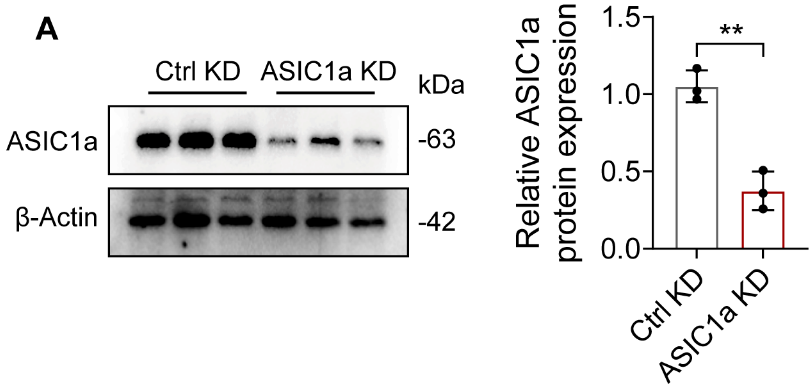

Figure S3. Validation of intra-articular AAV9-shASIC1a delivery and knockdown efficiency in rat cartilage. (A) Immunoblot analysis of ASIC1a in rats. Data were presented as mean ± SD and analyzed by Student's t-test. *p < 0.05, **p < 0.01, ***p < 0.001.

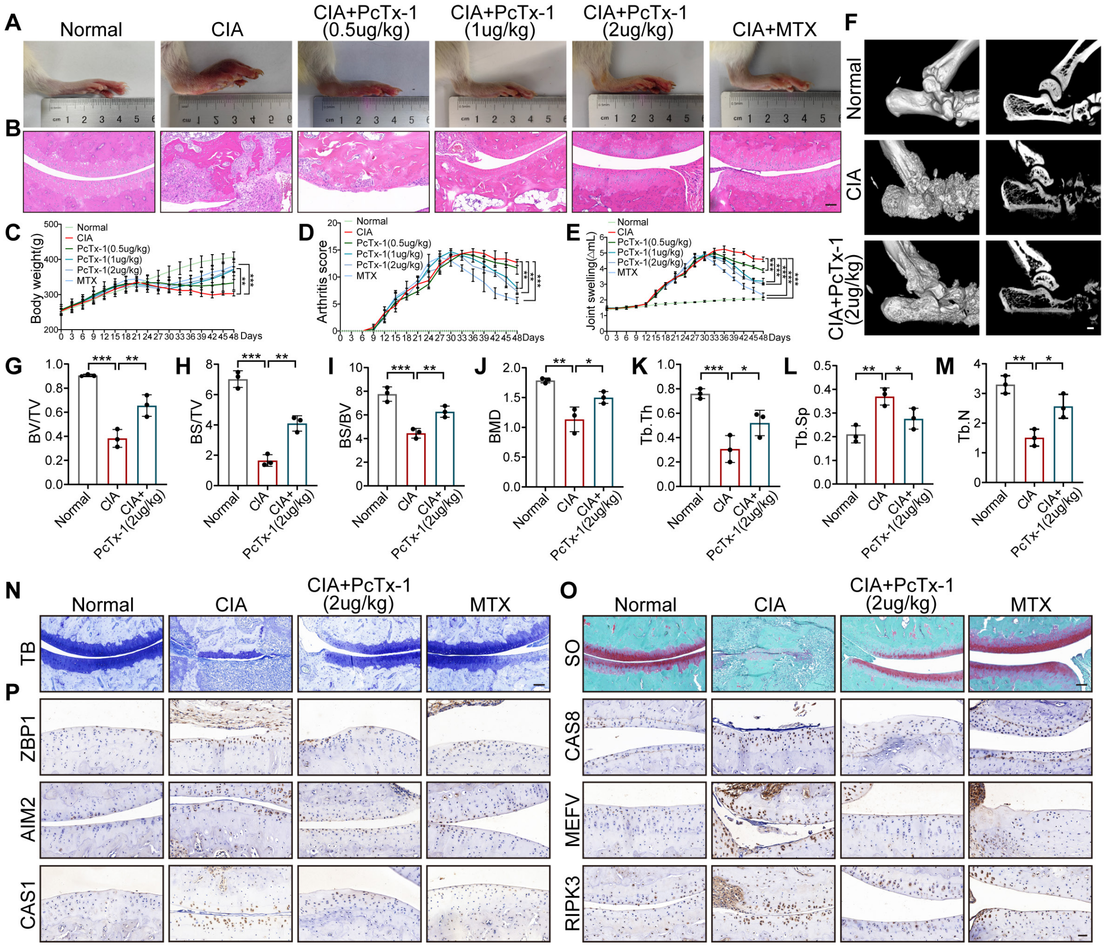

Figure S4. Pharmacological blockade of ASIC1a effectively improved CIA progression. (A) Representative images of ankle joints of rats (n = 8 per group). (B) Representative images of H&E staining of rat cartilages. Scale bar: 100 μm. (C) The body weight of rats. (D) The arthritis score of rats. (E) The joint swelling of rats. (F) Representative Micro-CT analysis of rat ankles. Scale bar: 1 mm. (G-M) Quantitative analysis of BV/TV, BS/TV, BS/BV, BMD, Tb.Th, Tb.Sp and Tb.N. (N-O) Representative images of toluidine blue and safranin O-fast green staining of rat cartilages. Scale bar: 100 μm. (P) Representative images of immunohistochemistry staining for ZBP1, AIM2, RIPK3, ASC, CAS8, CAS3, Pyrin and CAS1 in rat cartilages. Scale bar: 50 μm. Data were presented as mean ± SD and analyzed by Student's t-test or one-way ANOVA. *$p < 0.05$, **$p < 0.01$, ***$p < 0.001$.

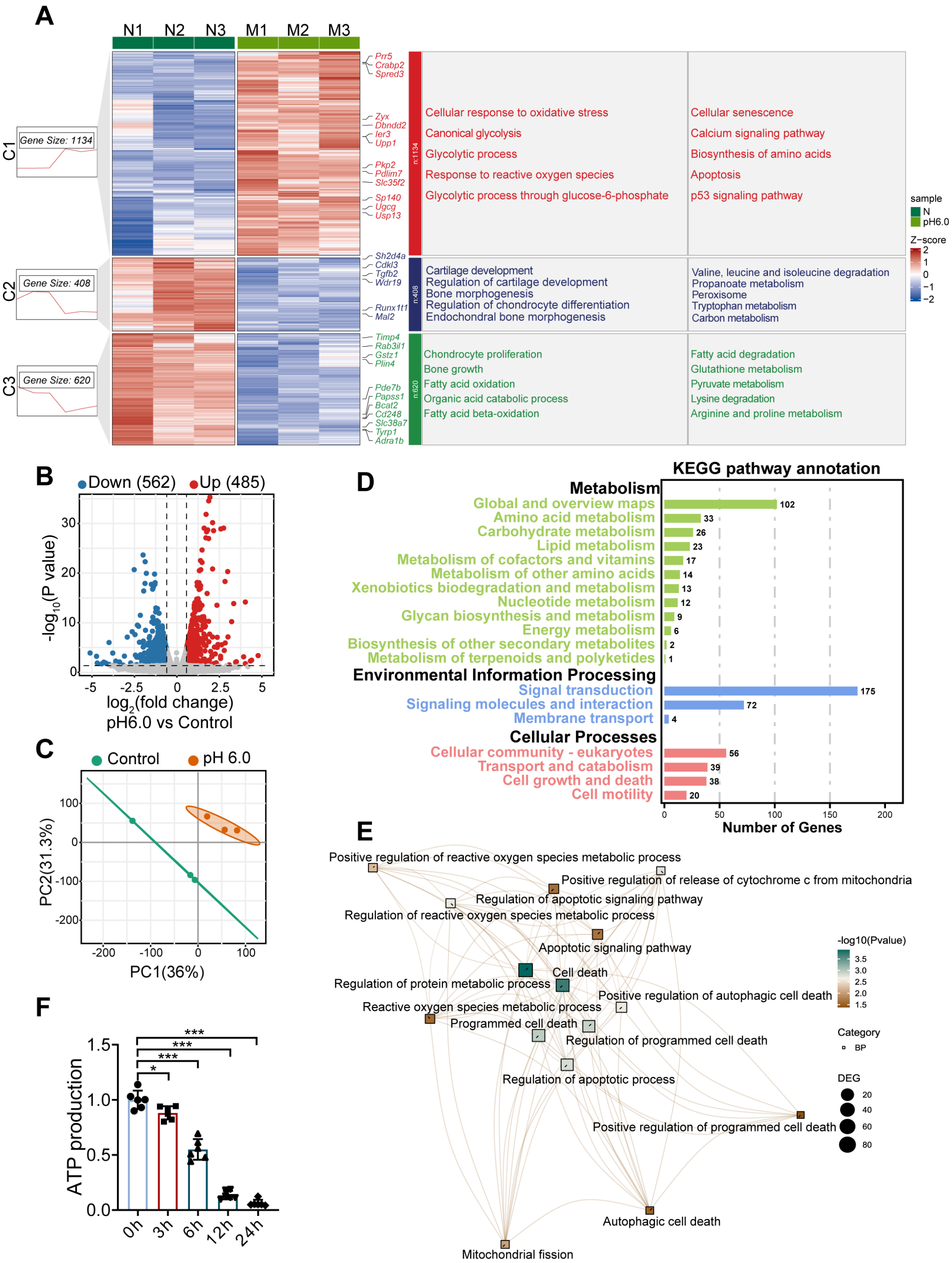

Figure S5. Chondrocyte RNA sequencing in pH6.0 acid group vs. control. (A) RNA-seq expression profiles clustered according to expression patterns, with clusters enriched by KEGG database and GO database. (B) Volcano plots showed the expression distribution of genes in the pH 6.0 group compared to the control group. (C) Principal component analysis showed the differential relationship between the pH 6.0 group and the control group. (D) Enrichment analysis of KEGG pathways between the pH 6.0 group and the control group. (E) Association network between

chondrocyte death and mitochondrial function. (F) The intracellular ATP production was measured using an ATP production assay Kit. Data were presented as mean ± SD and analyzed by Student's t-test or one-way ANOVA. *p < 0.05, **p < 0.01, ***p < 0.001.

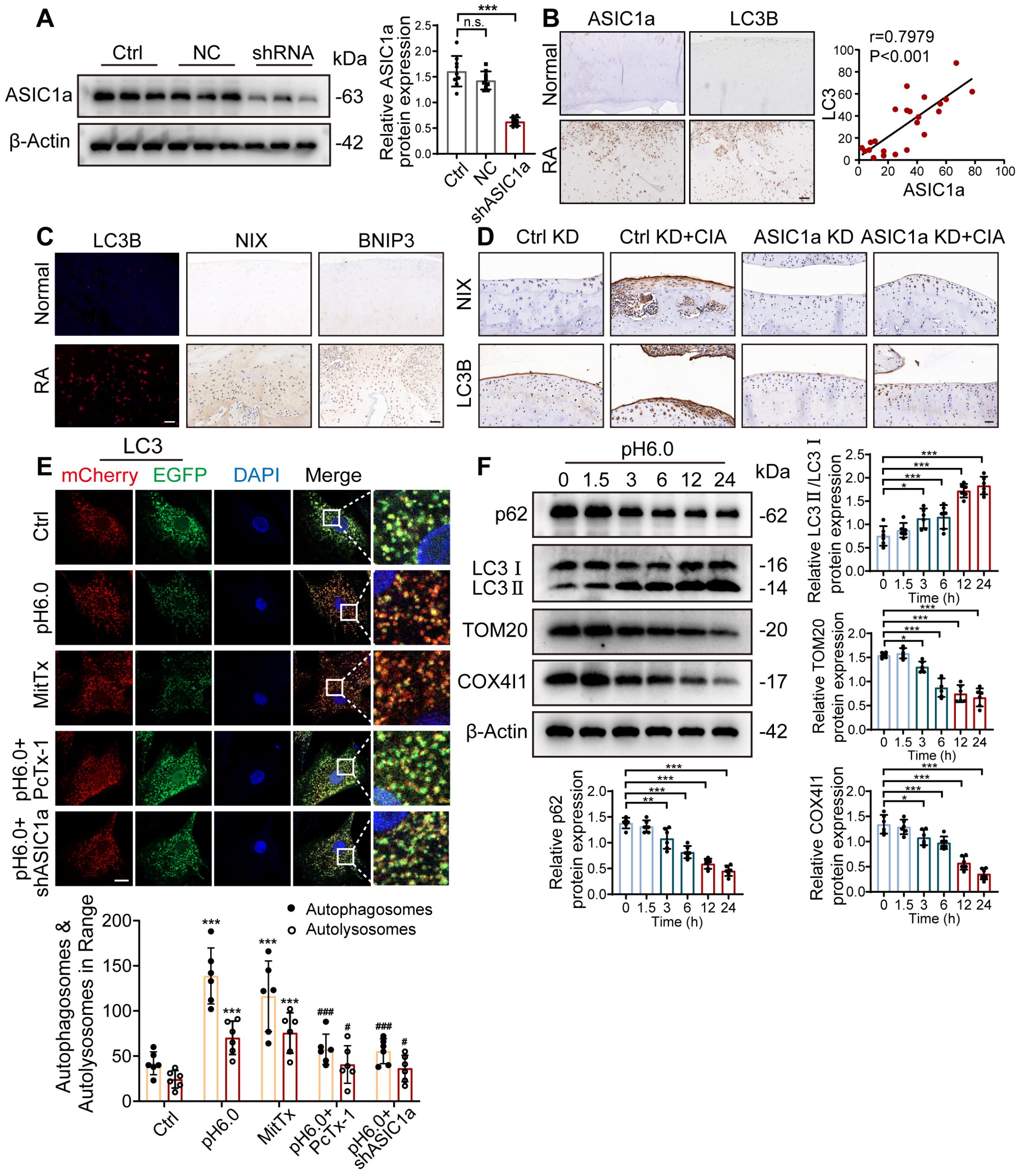

Figure S6. Mitophagy is associated with cartilage damage. (A) The ASIC1a and β-actin protein levels were determined by western blot in shASIC1a chondrocytes. (B) Immunohistochemistry staining for ASIC1a and LC3B in cartilage tissues from RA patients and the control group. Scale bar: 100 μm. (C) Immunofluorescence staining for LC3B and immunohistochemistry staining for BNIP3 and NIX in cartilage tissues from RA patients and the control group. Scale bar: 100 μm. (D) Representative immunohistochemistry staining images for NIX and LC3 in cartilages of rats. Scale bar: 500 μm. (E) Fluorescent dots transfected with LC3-EGFP-mCherry adenovirus in chondrocytes were observed by confocal microscopy. The number of yellow and red dots, which indicated the number of autophagosomes and autolysosome, was conducted. Data are representative of at least six independent experiments. Scale bar: 10 μm. (F) The TOM20, COX4I1, p62 and LC3 protein levels in chondrocytes were determined by western blot. Data were presented as mean ± SD and analyzed by

Student's t-test or one-way ANOVA. $*p < 0.05$, $**p < 0.01$, $***p < 0.001$.

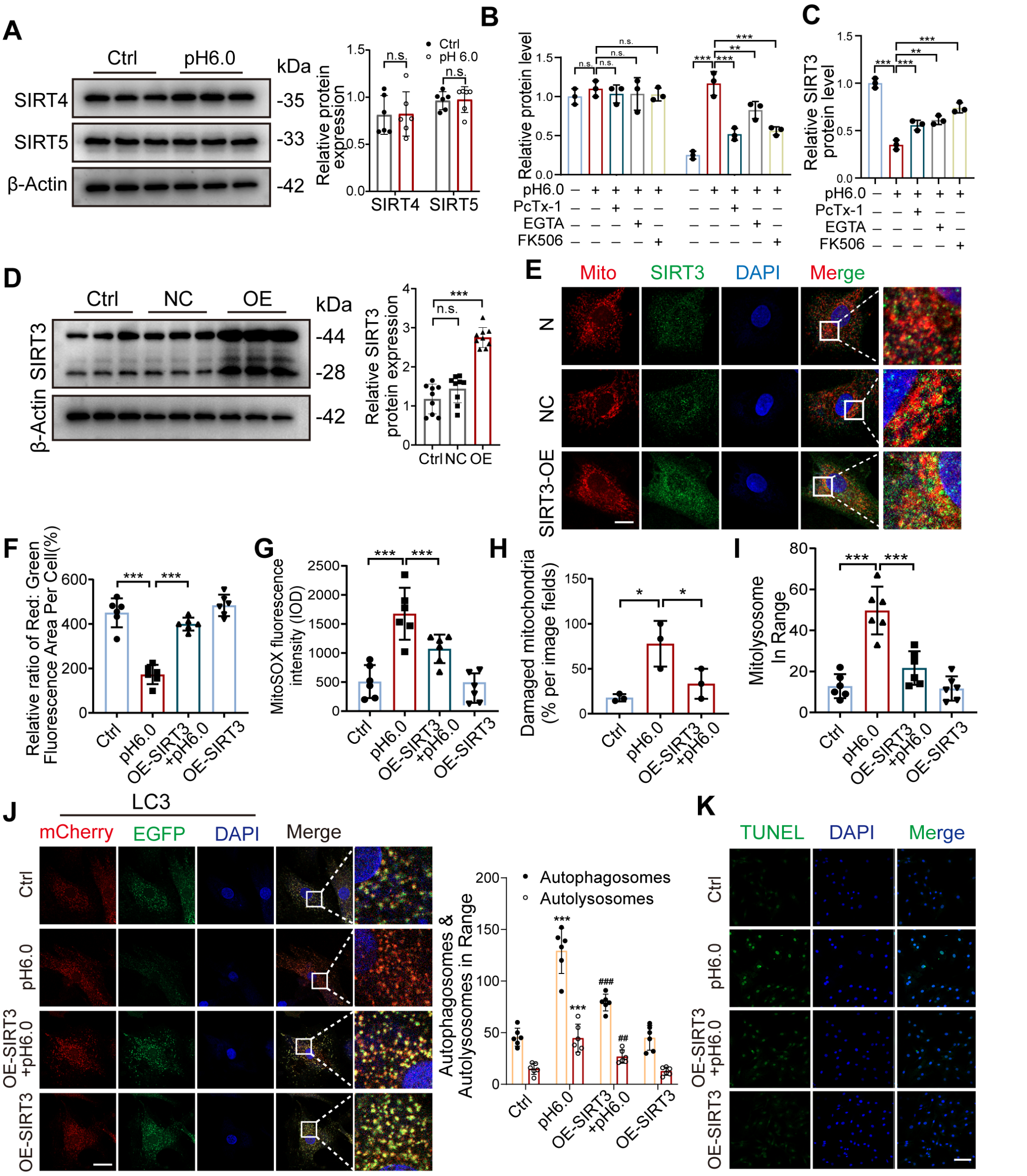

Figure S7. Restoration of SIRT3 inhibited excessive mitophagy and improved chondrocyte dysfunction. (A) The SIRT4 and SIRT5 protein levels were determined by western blot in chondrocytes. Data are representative of at least three independent experiments. (B) The protein levels of HSP70, CaN and β-actin in chondrocytes were determined by western blot. (C) The protein levels of SIRT3 and VDAC1 in chondrocytes were determined by western blot. (D) After overexpression of SIRT3, representative western blot bands of SIRT3 and β-actin. Data are representative of at least three independent experiments. (E) The colocalization of mitochondria and SIRT3 was observed. Scale bar: 10 μm. (F) The ratio of red to green fluorescence intensity was quantified to indicated the level of mitochondrial membrane potential. (G) Quantification of mitochondrial superoxide levels by fluorescence IOD analysis. (H) The number of damage mitochondria was determined. (I) The number of

mitolysosome was quantified. (J) Fluorescent dots transfected with LC3-EGFP-mCherry adenovirus in chondrocytes were observed by confocal microscopy. The number of yellow and red dots, which indicated the number of autophagosomes and autolysosome, was determined. Scale bar: 10 μm. (K) TUNEL assay was applied to detect apoptosis in chondrocytes. Images are representative of six independent experiments. Scale bar: 100 μm. Data were presented as mean ± SD and analyzed by Student's t-test or one-way ANOVA. *$p < 0.05$, **$p < 0.01$, ***$p < 0.001$.

Table S1. Sequences of AAV9-ASIC1a shASIC1a.

Gene	Forward primer sequence (5′ → 3′)	Reverse primer sequence (5′ → 3′)
ASIC1a-shRNA	CACCGCCAAGAAGTTCAACAAATCGTTCAAGAGACGATTTGTTGAACTTCTTGGCTTTTTTG	GATCCAAAAAAGCCAAGAAGTTCAACAAATCGTCTCTTGAACGATTTGTTGAACTTCTTGGC
NC-shRNA	CACCGTTCTCCGAACGTGTCACGTTTCAAGAGAACGTGACACGTTCGGAGAATTTTTTG	GATCCAAAAAATTCTCCGAACGTGTCACGTTCTCTTGAAACGTGACACGTTCGGAGAAC

Table S2. Antibody catalogue.

Name	Catalog No.	Source	Application/Dilution
TOMM20	ab186735	Abcam	WB/1:1000; IF/1:250
BNIP3	ab109362	Abcam	WB/1:1000; IF/1:150; IHC/1:200
NIX	ab109414	Abcam	WB/1:1000; IF/1:150; IHC/1:200
β-Actin	ab8226	Abcam	WB/1:1000
FUNDCL1	ab272627	Abcam	WB/1:1000
LC3B	ab192890	Abcam	WB/1:2000; IF/1:100; IHC: 1:100
p62	ab109012	Abcam	WB/1:10000
PARKIN	ab77924	Abcam	WB/1:2000
PINK	ab186303	Abcam	WB/1:1000
ASIC1a	ab300563	Abcam	WB/1:1000; IHC/1:100
ASIC1a	27235-1-AP	Proteintech	IF/1:100
Col2a1	ab307674	Abcam	WB/1:1000
MMP13	ab39012	Abcam	WB/1:3000
ASC	ab309497	Abcam	WB/1:1000; IF/1:500; IHC/1:100, IP/1:30
SIRT3	ab246522	Abcam	WB/1:1000
SIRT3	AF5135	Affinity	IF/1:200
ZBP1	13285-1-AP	Proteintech	WB/1:1000; IF/1:100; IHC/1:100
VDAC1	55259-1-AP	Proteintech	WB/1:1000; IF/1:100
Pyrin	24280-1-AP	Proteintech	WB/1:2000; IF/1:200; IHC/1: 100
AIM2	20590-1-AP	Proteintech	WB/1:1000; IF/1:20; IHC: 1:100
Caspase-1	22915-1-AP	Proteintech	WB/1:2000; IF/1:50; IHC/ 1: 100
c-Caspase-3	9661	Cell Signaling Technology	WB/1:2000; IF/1:400; IHC/ 1: 400
RIPK3	bs-3551R	Bioss	WB/1:500; IF/1:100; IHC/1: 100
Caspase-8	AF6442	Affinity	WB/1:1000; IF/1:100; IHC: 1:100

Table S3. Primer sequences for real-time PCR.

Gene	Forward primer sequence (5′ → 3′)	Reverse primer sequence (3′ → 5′)
β-actin	CCCATCTATGAGGGTTACGC	TTTAATGTCACGCACGATTTC
Sirt3	ACCCTGAGGCCATCTTTGAA	AAGCAGCCGAAGGAAGTAGT
Col2a1	CAGGGTGCTCGTGGATTCCC	TGGAGCACCAGCTTCTCCCT
Acan	GCTACCCTGATCCCTCATCC	GATGTCCTCTTCACCACCCA
Mmp13	GTGACAGGAGCTAAGGCAGA	AGCATGAAAGGGTGGTCTCA
Adamts	ACCGTTCCTGCAGTGTCATA	CAGGACACCTGCGTATTTGG
Nix	CGGCACAGAGACAGCACAGT	TGACGCCAGTGCTGACGAAG
Bnip3	AGCTGCCCTGCTACCTCTCA	GAACGCTGCTCCCATTCCCA
Pink	CCCACTGGACACACGACGTT	AGTGTGGGCATGGTGGCTTC

Table S4. Scoring system for subjective evaluation of arthritis severity.

Severity score	Degree of inflammation
0	No evidence of erythema and swelling
1	Erythema and mild swelling confined to the tarsals or ankle joint
2	Erythema and mild swelling extending from the ankle to the tarsals
3	Erythema and moderate swelling extending from the ankle to metatarsal joints
4	Erythema and severe swelling encompass the ankle, foot and digits, or ankylosis of the limb
